# Supplementary material for: Comparative humoral profiles in mpox cases, survivors, and vaccinated individuals reveal correlates of protection against MPXV
Source: Cell Rep Med. 2025 Dec 8;6(12):102483. doi: 10.1016/j.xcrm.2025.102483 (PMC12765839; doi:10.1016/j.xcrm.2025.102483)
Supplement: Document S1. Figures S1–S8 and Table S2 [file mmc1.pdf]

Cell Reports Medicine, Volume *6*

Supplemental information

Comparative humoral profiles in mpox cases, survivors, and vaccinated individuals reveal correlates of protection against MPXV

Yanqun Wang, Lu Zhang, Lijuan Zhou, Jiantao Chen, Zhaoyong Zhang, Tiantian Wu, Peilan Wei, Airu Zhu, Ruoxi Cai, Jingjun Zhang, Zhiwei Lin, Canjie Chen, Yuanyuan Zhang, Qier Zhong, Jing Sun, Yongxia Shi, Jingxian Zhao, Jun Dai, Pengzhe Qin, and Jincun Zhao

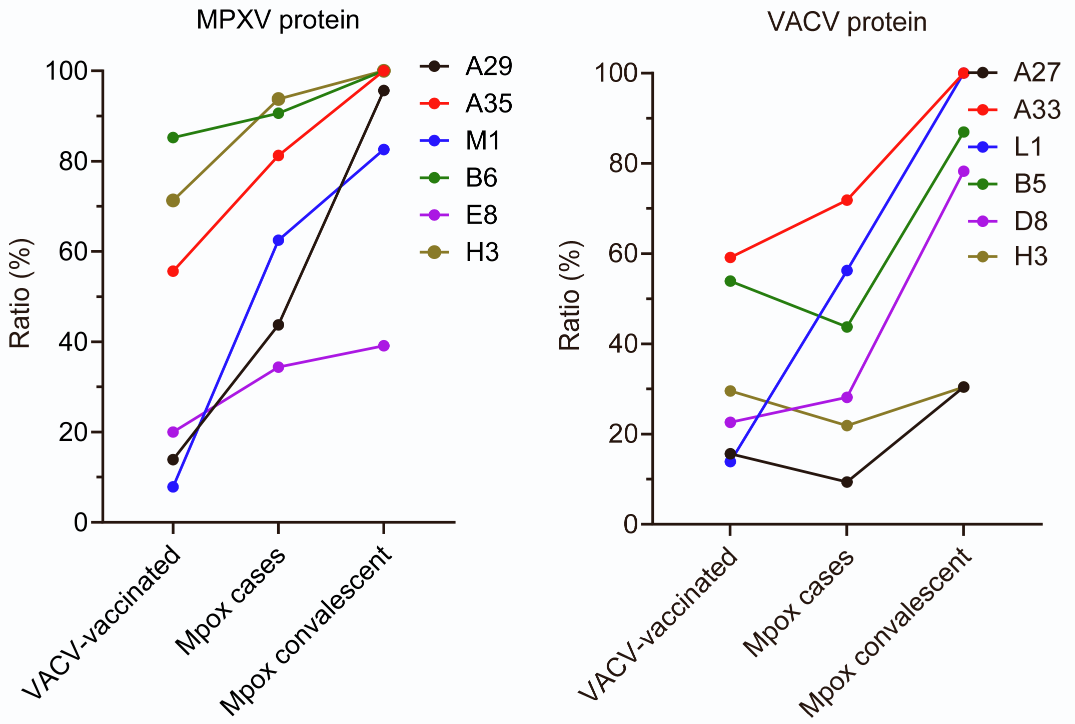

Figure S1. Comparisons of seropositivity rates of binding IgG against different orthopoxvirus antigens between mpox cases, convalescents and VACV vaccinated individuals. Related to Figure 1.

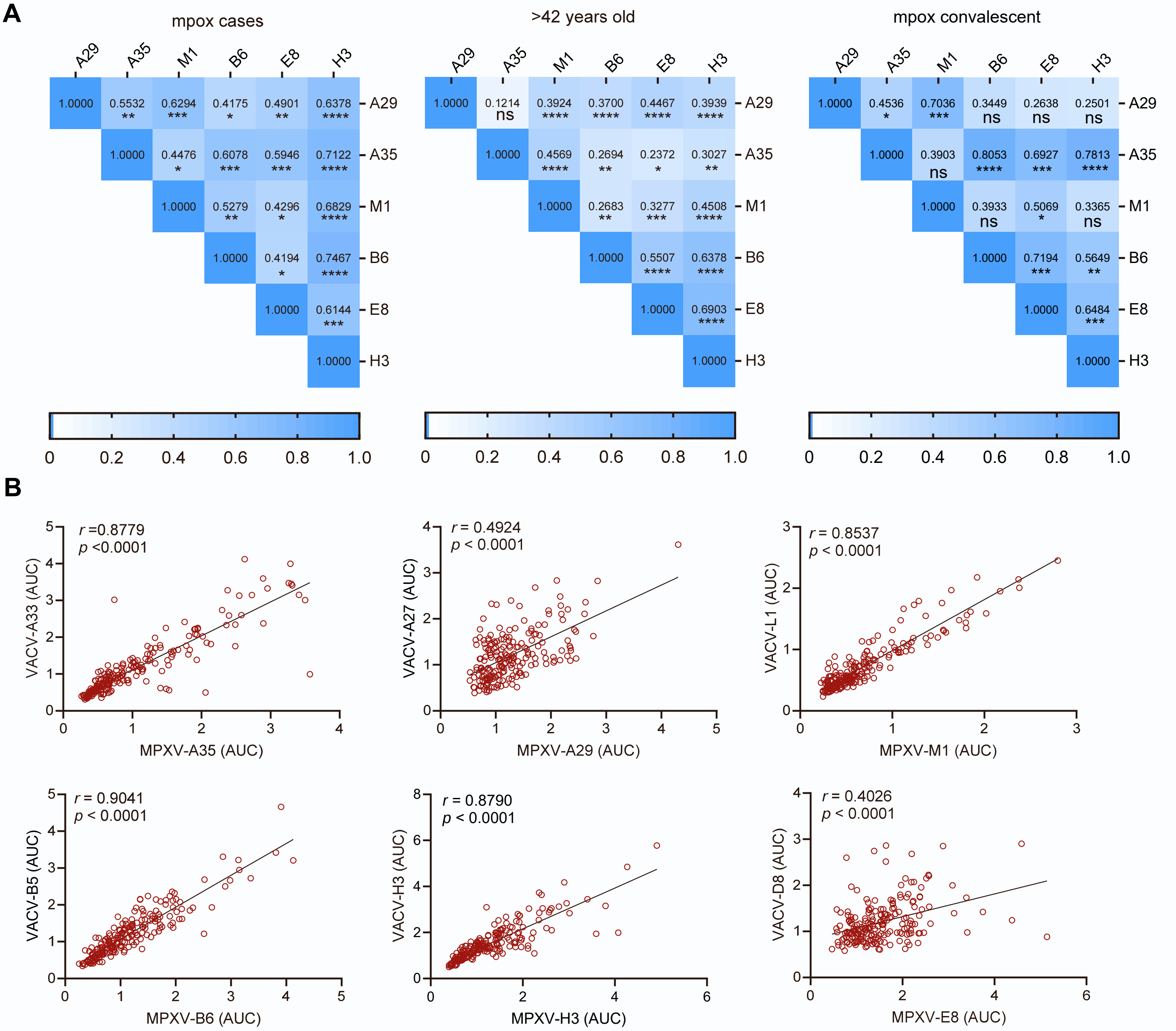

Figure S2. Correlation analysis of MPXV- and VACV-specific protein binding IgG response. Related to Figure 1. **(A)** Correlation matrix of IgG antibody response against different MPXV antigens (A29, A35, M1, B6, E8 and H3) in mpox cases, convalescents and VACV-vaccinated individuals (>42 years old). Color scale represents the value of the correlation between two variables. **(B)** Correlation analysis of MPXV (A29, A35, M1, B6, E8 and H3) and VACV (A33, A27, L1, B5, D8 and H3) antigens binding IgG response among all participants. Spearman's correlation coefficients and *p* values are shown.

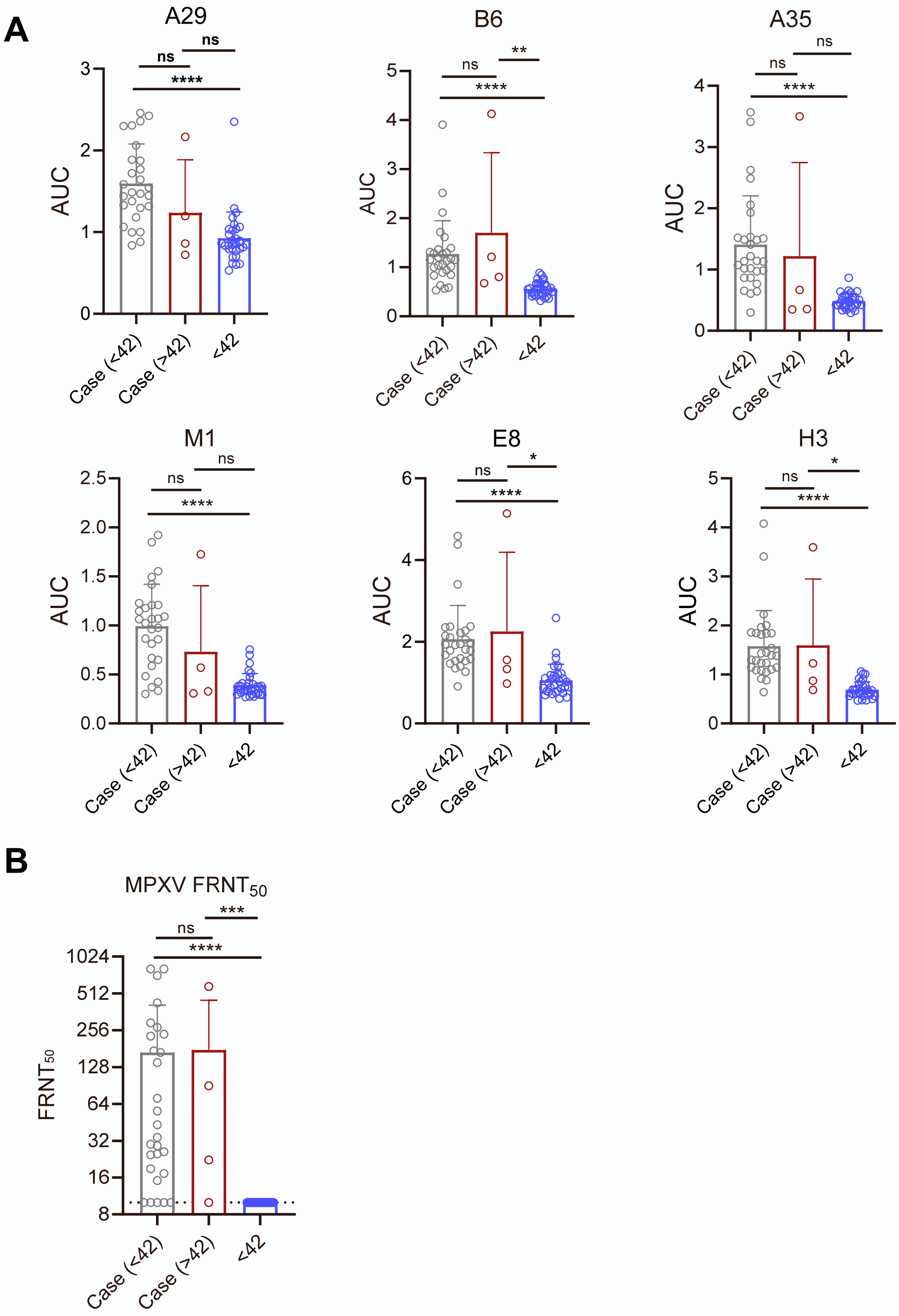

Figure S3. Pre-existing humoral immunity to orthopoxvirus does not impact the MPXV antibody response based on the limited participants. Related to Figure 1. **(A)** Comparisons of seropositivity rates of binding IgG against different MPXV antigens (A29, A35, M1, B6, E8 and H3) between mpox cases with and without pre-existing VACV antibody. **(B)** Comparisons of neutralizing antibody titer against authentic MPXV between mpox cases with (case with age >42) and without pre-existing VACV antibody (case with age <42). Statistical significance was assessed by one-way ANOVA with multiple comparisons in (A) and (B). Error bars represent mean ± SD, *p*-values are displayed as ns for $p > 0.05$, *$p < 0.05$, **$p < 0.01$ and ***$p < 0.001$.

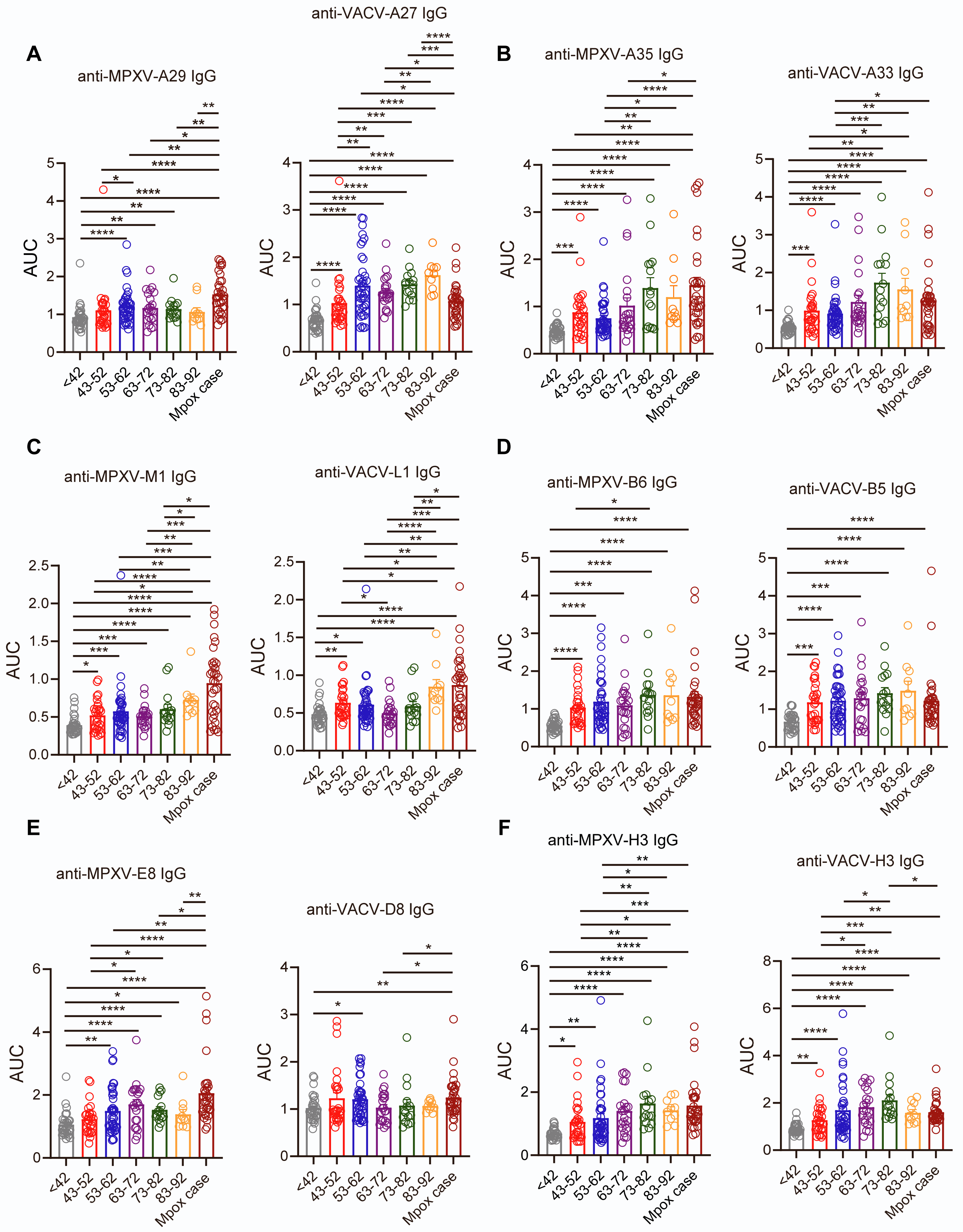

Figure S4. The distribution of antibody titers against multiple orthopoxvirus antigens (A-F) was analyzed in VACV-vaccinated donors, stratified into five age groups by decade of birth. Related to Figure 2. VACV-unvaccinated individuals and MPXV-infected cases were included as controls

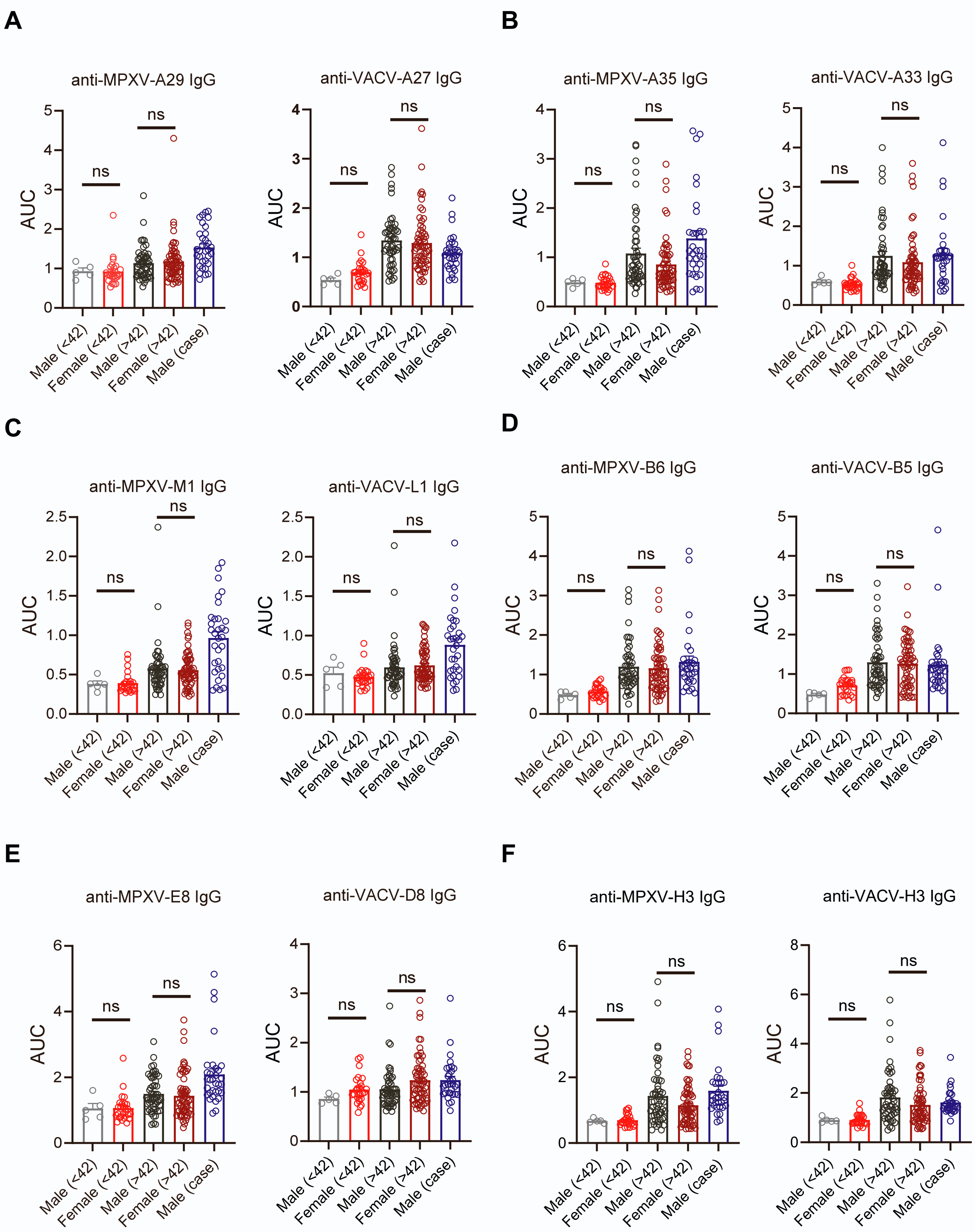

Figure S5. Relationship between binding antibodies against orthopoxvirus antigens and gender. Related to Figure 2. (A-F) Comparison of IgG antibody response against MPXV (A29, A35, M1, B6, E8, H3)) and VACV (A27, A33, L1, B5, D8, H3) antigens across different gender cohorts. Statistical significance was assessed using one-way ANOVA with multiple comparisons tests. Error bars represent mean ± SD, p-values are displayed as ns for $p > 0.05$, $*p < 0.05$, $**p < 0.01$ and $***p < 0.001$.

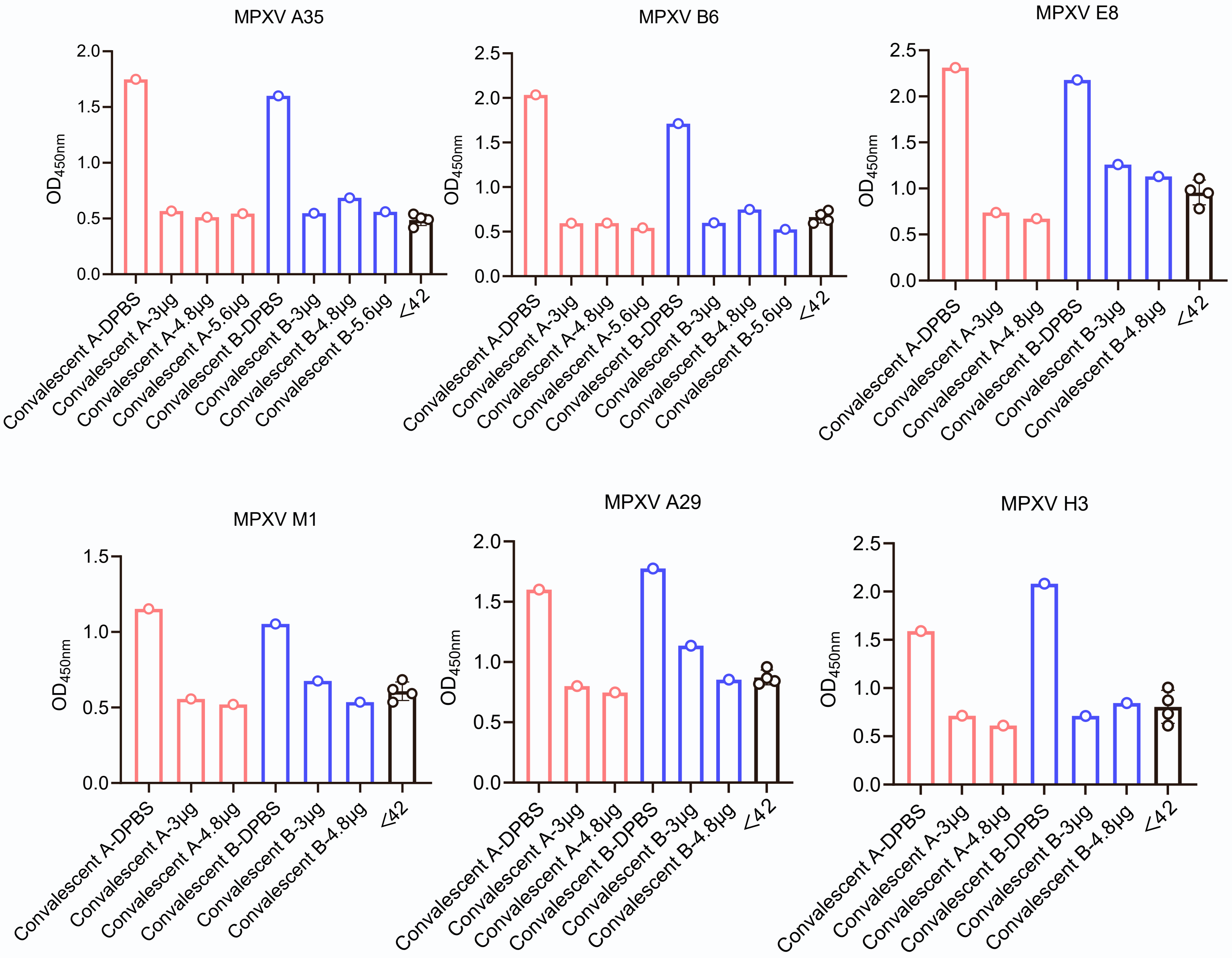

Figure S6. Quantification of plasma IgG binding to MPXV membrane proteins following competitive blocking with increasing amounts of membrane protein. Related to Figure 5. Plasma from two mpox convalescents (A and B) and four VACV-unvaccinated individuals (controls) was analyzed. Six membrane proteins (A35, B6, E8, M1, A29, and H3) were included in the assay. Data are representative of two independent experiments.

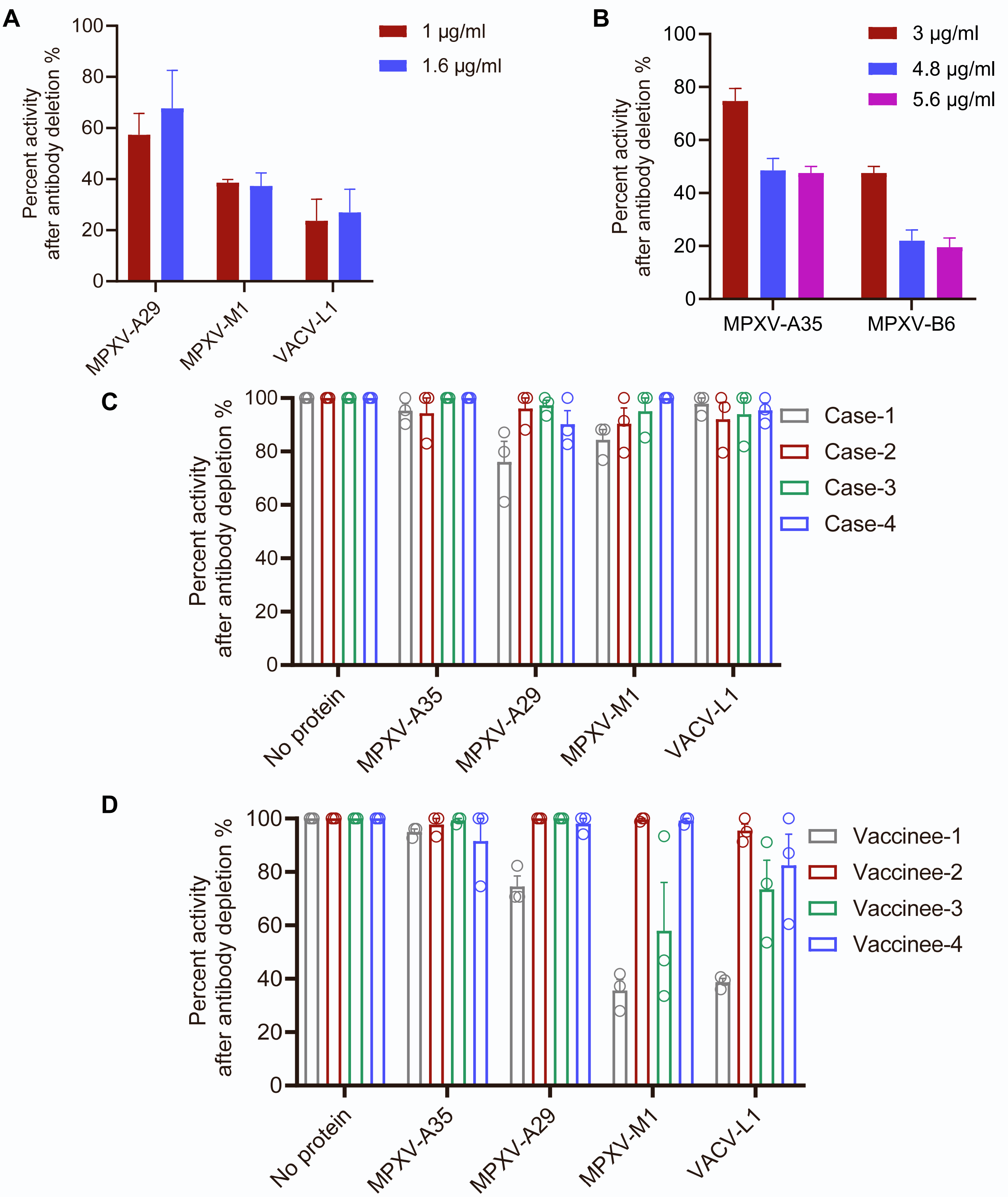

Figure S7. Optimization and application of antibody depletion assays. Related to Figure 5. (**A–B**) Optimization of antigen concentrations for antibody depletion. Residual neutralizing activity against IMV (**A**) and EEV (**B**) in human plasma following depletion with varying antigen concentrations. Based on these results, protein concentrations of 1 μg/mL (IMV) and 4.8 μg/mL (EEV) were selected for subsequent assays. (**C–D**) Application of antibody depletion assays. Residual neutralizing activity against MPXV IMV after depletion with purified A35, A29, M1, or L1 proteins in plasma from MPXV-infected individuals (**C**) and historically VACV-vaccinated individuals (**D**). Antibody depletion assays targeting IMV membrane proteins were evaluated using FRNT, whereas those targeting EEV membrane proteins were assessed using PRNT.

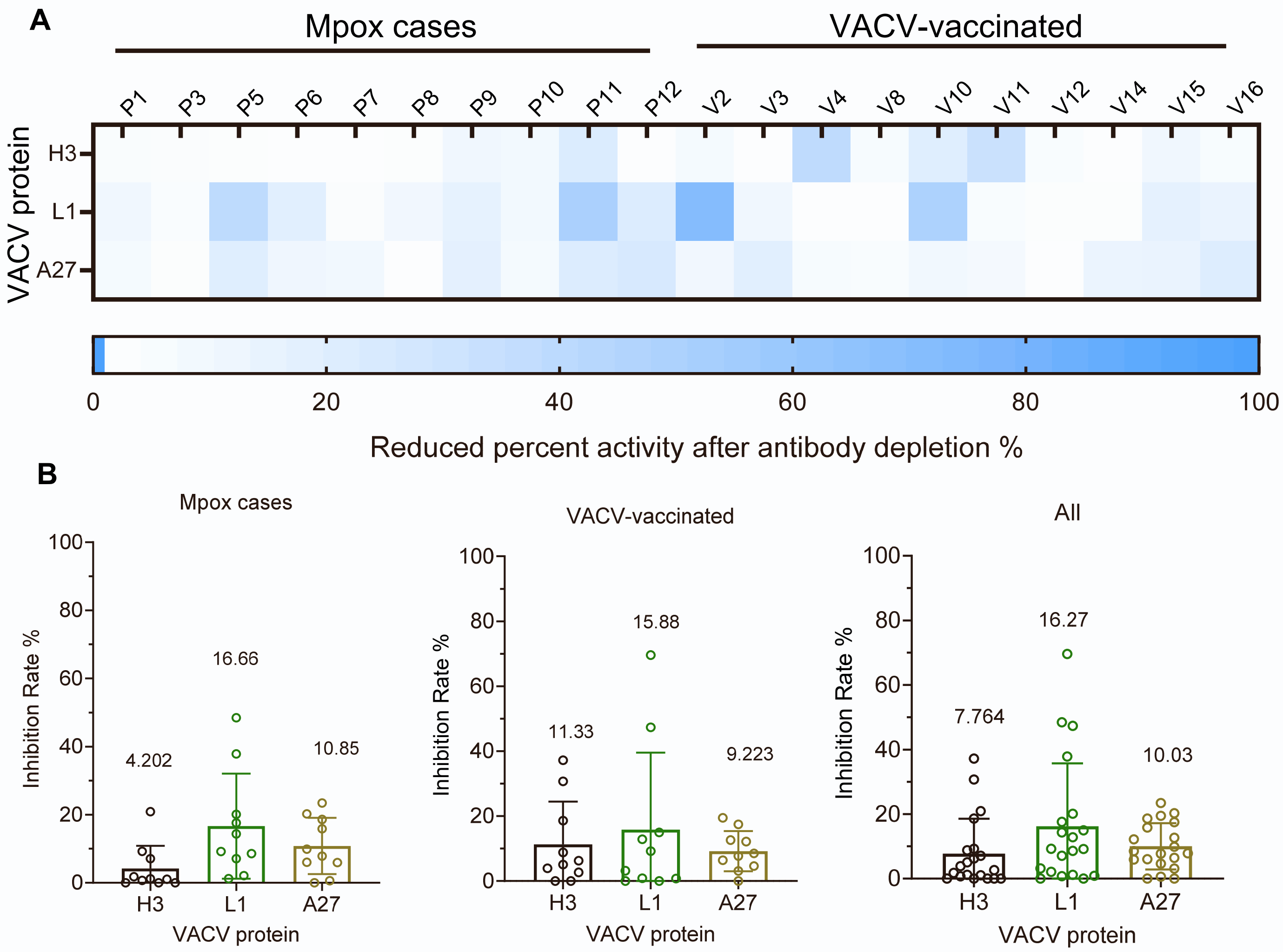

Figure S8. Quantification of neutralizing antibody responses against representative antigens of VACV by antibody depletion experiments. Related to Figure 5. (A) Heatmap of reduced neutralizing activity percentage of plasma after antibody depletion from VACV-vaccinated (>42 years old) or MPXV-infected individuals using multiple antigens of VACV (H3, L1 and A27). Donors' number was labeled in every column. Color scale represents the percentage of reduced neutralizing activity against VACV IMV. **(B)** Comparison of reduced neutralizing activity against IMV after incubation with variable VACV antigens. The average inhibition ratios are shown in the upper panel.

Table S2. Neutralizing titers against MPXV were determined from pooled plasma samples. Related to Figure 6.

MPXV $FRNT_{50}$	< 42	> 42	Case	Convalescent
Complement (-)	10	65.16	288.4	138.03
Complement (+)	10	173.78	401.79	325.83
