# Supplementary material for: Comparative humoral profiles in mpox cases, survivors, and vaccinated individuals reveal correlates of protection against MPXV
Source: Cell Rep Med. 2025 Dec 8;6(12):102483. doi: 10.1016/j.xcrm.2025.102483 (PMC12765839; doi:10.1016/j.xcrm.2025.102483)
Supplement: Document S2. Article plus supplemental information [file mmc3.pdf]

Cell Reports Medicine

Article

Comparative humoral profiles in mpox cases, survivors, and vaccinated individuals reveal correlates of protection against MPXV

Graphical abstract

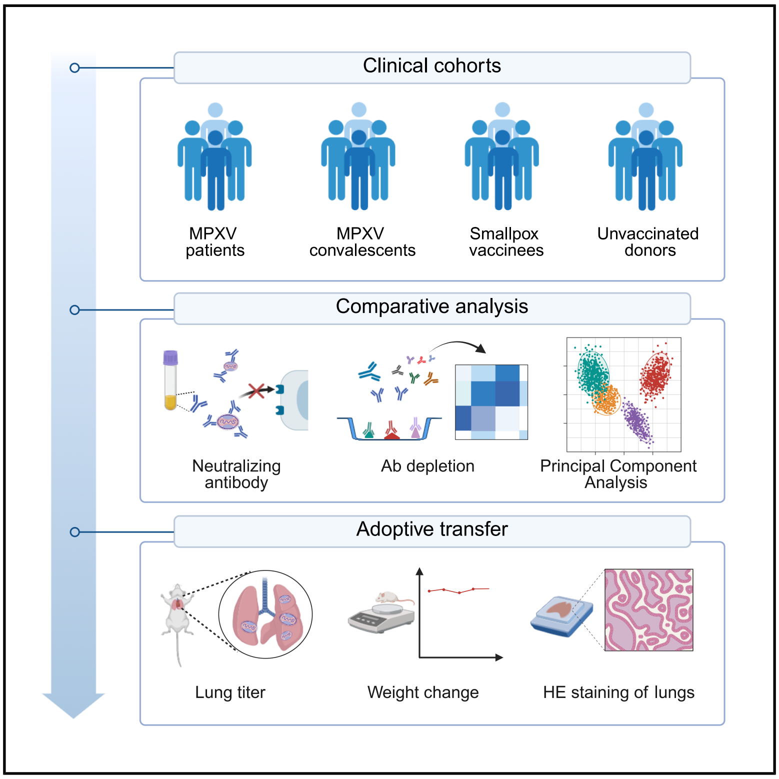

Authors

Yanqun Wang, Lu Zhang, Lijuan Zhou, ..., Jun Dai, Pengzhe Qin, Jincun Zhao

Correspondence

wangyanqun@gird.cn (Y.W.),
zhaojingxian@gird.cn (J.Z.),
daij@iqtcnet.cn (J.D.),
petgyy@gmail.com (P.Q.),
zhaojincun@gird.cn (J.Z.)

In brief

Wang et al. compare humoral profiles in mpox patients, convalescents, and vaccinated individuals, identifying dominant MPXV-neutralizing targets and defining antigenic and demographic correlates of orthopoxvirus immunity. Infection- and vaccination-induced antibodies confer *in vivo* protection, revealing determinants of effective humoral defense against MPXV.

Highlights

- Comprehensive profiling defines humoral correlates of protection to MPXV
- Serology shows age-related differences in binding and neutralizing titers post-VACV
- Antibody depletion reveals IMV and EEV neutralization hierarchies
- Passive transfer of MPXV-immune plasma suppresses viral replication *in vivo*

Wang et al., 2025, Cell Reports Medicine *6*, 102483
December 16, 2025 © 2025 The Author(s). Published by Elsevier Inc.
https://doi.org/10.1016/j.xcrm.2025.102483

Cell Reports Medicine

CellPress
OPEN ACCESS

Article

Comparative humoral profiles in mpox cases, survivors, and vaccinated individuals reveal correlates of protection against MPXV

Yanqun Wang,[1,8,*] Lu Zhang,[2,7] Lijuan Zhou,[3,4,7] Jiantao Chen,[1,7] Zhaoyong Zhang,[1,7] Tiantian Wu,[2] Peilan Wei,[1,5] Airu Zhu,[1] Ruoxi Cai,[1] Jingjun Zhang,[1] Zhiwei Lin,[2] Canjie Chen,[1] Yuanyuan Zhang,[1] Qier Zhong,[1,6] Jing Sun,[1] Yongxia Shi,[2] Jingxian Zhao,[1,5,*] Jun Dai,[1,2,5,*] Pengzhe Qin,[3,4,*] and Jincun Zhao[1,5,7,9,*]

[1]State Key Laboratory of Respiratory Disease, National Clinical Research Center for Respiratory Disease, Guangzhou Institute of Respiratory Health, the First Affiliated Hospital of Guangzhou Medical University, Guangzhou, China

[2]Health and Quarantine Laboratory, State Key Laboratory of Respiratory Disease of Guangzhou Customs District Technology Center, Guangzhou, China

[3]Guangzhou Center for Disease Control and Prevention (Guangzhou Health Supervision institute), Guangzhou, China

[4]Institute of Public Health, Guangzhou Medical University & Guangzhou Center for Disease Control and Prevention (Guangzhou Health Supervision institute), Guangzhou, China

[5]Guangzhou National Laboratory, Guangzhou, China

[6]GMU-GIBH Joint School of Life Sciences, Guangzhou Medical University, Guangzhou, China

[7]Shanghai Institute for Advanced Immunochemical Studies, School of Life Science and Technology, ShanghaiTech University, Shanghai, China

[8]These authors contributed equally

[9]Lead contact

*Correspondence: wangyanqun@gird.cn (Y.W.), zhaojingxian@gird.cn (J.Z.), daij@iqtcnet.cn (J.D.), petgyy@gmail.com (P.Q.), zhaojincun@gird.cn (J.Z.)

https://doi.org/10.1016/j.xcrm.2025.102483

SUMMARY

Mpox is regarded as the most important orthopoxvirus infection since the eradication of smallpox, yet the determinants of protective antibody responses remain poorly defined. Here, we investigate factors shaping humoral protection against MPXV and observe a moderate correlation between orthopoxvirus antibody levels and age among vaccinia virus (VACV)-vaccinated individuals. No correlation is found between orthopoxvirus-binding antibodies and gender. Pre-existing humoral immunity does not impair the induction of MPXV-specific antibody responses in mpox cases. Despite a high seropositivity rate for neutralizing antibodies in both mpox cases and vaccinated individuals, the overall titers remain modest. Quantitative analyses identify MPXV antigens A29, E8, and M1 on intracellular mature virion (IMV), together with A35 and B6 on extracellular enveloped virion (EEV), as the principal targets of neutralizing antibodies elicited during both MPXV infection and vaccination. Passive transfer of plasma from mpox patients and convalescents significantly reduces viral replication *in vivo*. These findings provide critical insights into the correlates of protection against MPXV.

INTRODUCTION

Mpox, caused by the MPXV, has re-emerged as a global health concern following large-scale outbreaks in 2022 and 2024. These events prompted the World Health Organization (WHO) to declare a Public Health Emergency of International Concern.[1,2] MPXV belongs to the genus *Orthopoxvirus*, which also includes variola virus, vaccinia virus (VACV), and cowpox virus.[3] Infection or immunization with one orthopoxvirus (OPXV) generally confers cross-protection against the others.[4–7] However, the cessation of universal smallpox vaccination programs in the 1970s has led to a gradual decline in herd immunity, potentially facilitating the resurgence of MPXV.[8,9] Moreover, smallpox vaccination does not provide complete protection against MPXV during the current outbreak.[10,11]

Defining the determinants of protective antibody responses to MPXV is critical for informing vaccination strategies and public health policy. Neutralizing antibodies (nAbs) induced by smallpox vaccination can persist for decades and confer partial cross-protection against MPXV.[12,13] However, the degree of protection varies depending on the vaccine strain and time since immunization.[14] Older cohorts vaccinated prior to 1981 generally retain neutralizing activity, whereas younger, unvaccinated populations lack such immunity.[15] This heterogeneous immunological landscape complicates assessment of population-level protection and underscores the need to define correlates of antibody-mediated protection.

© 2025 The Author(s). Published by Elsevier Inc.
This is an open access article under the CC BY license (http://creativecommons.org/licenses/by/4.0/).

Table 1. The characteristic information of participants in this study

Sample group (birth year)	Number	Age range	Percentage (%)
VACV unvaccinated 1982–1999 (<42 years)	31	24–41	100% (31/31)
VACV vaccinated 1931–1981 (>42 years)	28	43–52	24.3% (28/115)
	40	53–62	34.8% (40/115)
	22	63–72	19.1% (22/115)
	15	73–82	13.0% (15/115)
	10	83–92	8.7% (10/115)
mpox cases	28	<42	87.5% (28/32)
	4	>42	12.5% (4/32)
mpox convalescents (6–9 m.p.i)	22	<42	95.6% (22/23)
	1	>42	4.4% (1/23)

m.p.i, months post-infection.

Recent serological studies have begun to characterize neutralizing responses elicited by both MPXV infection and smallpox vaccination.[15,16] However, several gaps remain in our understanding, with one of the major challenges being the diversity of antigenic proteins expressed by MPXV, which complicates the identification of key protective antigens. Moreover, the cross-reactivity of antibodies between MPXV and other OPXV raises concerns about the specificity and efficacy of these responses, especially in populations with heterogeneous immunological profiles. Moreover, recent research indicated that while smallpox vaccines provide partial clinical protection, the levels of MPXV nAbs in vaccinated individuals remain relatively low.[17–19]

Accordingly, identifying humoral correlates of protection, including pre-existing immunity, neutralizing titers, age, and gender, is essential for optimizing vaccination strategies. Here, we performed an integrated analysis of antibody responses in acute mpox patients, convalescents, and historically VACV-vaccinated individuals across diverse age cohorts, combining serology, antigen-specific profiling, and *in vivo* validation.

RESULTS

Participants enrolled in the study

A total of 32 plasma samples were collected in 2023 from MPXV-infected individuals (age range: 18–45 years) diagnosed at the Guangzhou Center for Disease Control and Prevention. MPXV infection was confirmed by PCR testing of swabs from skin lesions. All patients were sampled at a single time point, between 4 and 10 days after symptom onset. Most patients (87.5%, 28/32) were under 42 years of age and reported no history of smallpox vaccination. An additional 23 plasma samples were obtained from mpox-convalescent individuals 6–9 months post-infection. All individuals, whether in the acute or convalescent phase, exhibited mild disease, and no longitudinal sampling was performed (Table 1).

In parallel, 146 healthy individuals were randomly selected and stratified into two age-based cohorts according to the historical smallpox vaccination policy in China. Thirty-one unvaccinated individuals born after 1982 served as negative controls, while 115 individuals born before 1981 were presumed to have received childhood smallpox vaccination with VACV, which was mandatory until that year. The vaccinated cohort was further subdivided into five age groups: 43–52, 53–62, 63–72, 73–82, and 83–92 years. Participant demographics, including age and gender, are detailed in Tables 1 and S1. Together, these cohorts provided a comprehensive framework for comparative serological analyses.

A35, M1, B6, and H3 proteins were preferentially recognized by MPXV-specific antibody in patients and convalescents

To compare the antigenicity of MPXV and VACV proteins, we established in-house ELISAs targeting 12 major surface proteins (MPXV: A29, A35, M1, B6, E8, and H3; VACV: A27, A33, L1, B5, D8, and H3). Distinct IgG profiles were observed across unvaccinated controls, historically VACV-vaccinated individuals, acute mpox cases, and convalescents (Figure 1). In acute infection, seroconversion rates to A35, M1, B6, and H3 reached 62%–93% by 4–10 days after onset, increasing to 82%–100% during convalescence. By contrast, seroconversion to MPXV E8 was markedly lower (34%–39%), indicating that only a subset of proteins contribute robustly to the early antibody response. ELISAs based on A35, M1, B6, and H3 displayed superior diagnostic sensitivity, detecting MPXV-specific antibodies in the majority of cases and convalescents. Additionally, results indicated that plasma from both VACV-vaccinated individuals and MPXV patients shared a similar trend and showed high levels of cross-reactivity against both VACV and MPXV main surface proteins, as predicted, since VACV and MPXV share 93.64%–98.8% amino acid homology in their main surface proteins (Figure 1G).

Distinct from the MPXV serological profile, the VACV A33 and B5 proteins were preferentially recognized in vaccinated individuals (Figure S1). Specific IgG antibodies against VACV proteins were detected in less than 60% of vaccine recipients, suggesting that while a subset of VACV antigens are still recognized, there has been a noticeable decline in recognition over time. Furthermore, the ratio of antibody binding to the MPXV M1 and its VACV homolog L1 varies between vaccinated individuals and MPXV patients, highlighting antigenic diversity in the humoral response to OPXVs.

Correlation analysis further showed that IgG responses to MPXV proteins (A29, A35, B6, E8, M1, and H3) were moderately ($0.3 < r < 0.5$) to strongly ($0.5 < r < 1.0$) correlated with each other (Figure S2A). Binding titers to MPXV proteins also correlated significantly with those to their VACV homologs in both vaccinated donors and mpox patients (Figure S2B), highlighting the extensive cross-reactivity within the *Orthopoxvirus* genus.

Pre-existing humoral immunity does not influence the induction of MPXV-specific antibody responses in mpox cases

A key unresolved question is whether prior immunity to OPXV influences infection or vaccine-induced immunity. We compared the IgG response between MPXV-infected patients with a history of VACV vaccination and those without vaccination. While studies have identified cross-reactive antibodies that bind both MPXV and VACV, there remains significant controversy regarding whether VACV antibodies are boosted following

CellPress
OPEN ACCESS

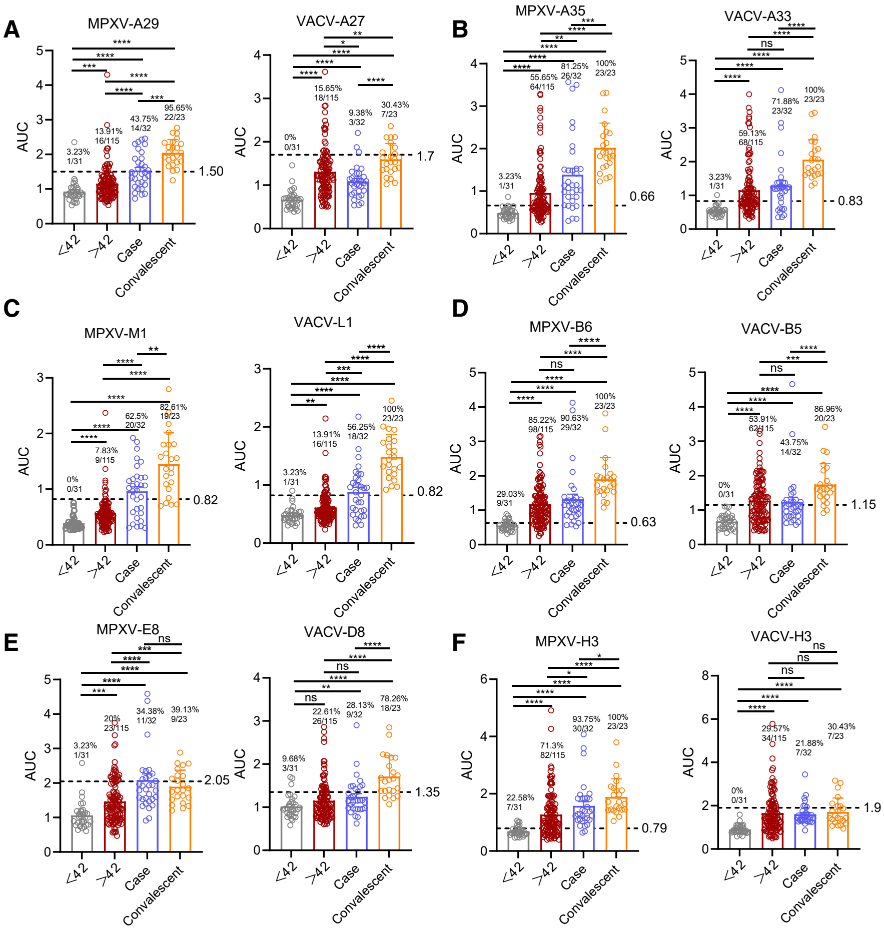

G

MPXV protein	Ratio (<42)	Ratio (>42)	Ratio (cases)	Ratio (convalescents)	VACV protein	Ratio (<42)	Ratio (>42)	Ratio (cases)	Ratio (convalescents)	AA homology
A29	3.23%	13.91%	43.75%	95.65%	A27	0%	15.65%	9.38%	30.43%	93.64%
A35	3.23%	55.65%	81.25%	100%	A33	3.23%	59.13%	71.88%	100%	94.05%
M1	0%	7.83%	62.5%	82.61%	L1	3.23%	13.91%	56.25%	100%	98.80%
B6	29.03%	85.22%	90.63%	100%	B5	0%	53.91%	43.75%	86.96%	95.90%
E8	3.23%	20%	34.38%	39.13%	D8	9.68%	22.61%	28.13%	78.26%	94.75%
H3	22.58%	71.3%	93.75%	100%	H3	0%	29.57%	21.88%	30.43%	94.44%

Figure 1. Differential antibody responses against orthopoxvirus after historic VACV vaccination and MPXV infection

(A–F) Characterization of IgG antibody response against MPXV (A29, A35, M1, B6, E8, and H3) and VACV (A27, A33, L1, B5, D8, and H3) antigens in four cohorts, including unvaccinated cohort (<42 years, $n = 31$), VACV-vaccinated cohort (>42 years, $n = 115$), mpox cases ($n = 32$) and convalescents ($n = 23$). AUCs of binding IgG responses against multiple orthopoxvirus antigens were compared among different populations. The limit of detection (LOD) was determined using samples from the unvaccinated cohort (individuals under 20 years of age), defined as the mean plus 3 SDs. Seropositivity rates of IgG antibody were indicated above the bar.

(G) Summary of seropositivity rates of IgG antibody against MPXV and VACV antigens. Comparative amino acid homology analyses were performed to assess the similarity between MPXV and VACV major surface proteins.

Statistical significance was assessed using one-way ANOVA with multiple comparisons tests. The data comprise 3 independent biological replicates, with datasets including 2–3 technical replicates. Error bars represent mean ± SD, p values are displayed as ns for $p > 0.05$, *$p < 0.05$, **$p < 0.01$, ***$p < 0.001$, and ****$p < 0.0001$.

MPXV infection. Our results indicated that pre-existing VACV antibodies do not appear to influence the MPXV antibody response (Figure S3), and no notable differences in seropositivity rates of binding and nAbs against MPXV were observed between mpox cases with or without pre-existing VACV antibodies. However, given the limited number of participants, further

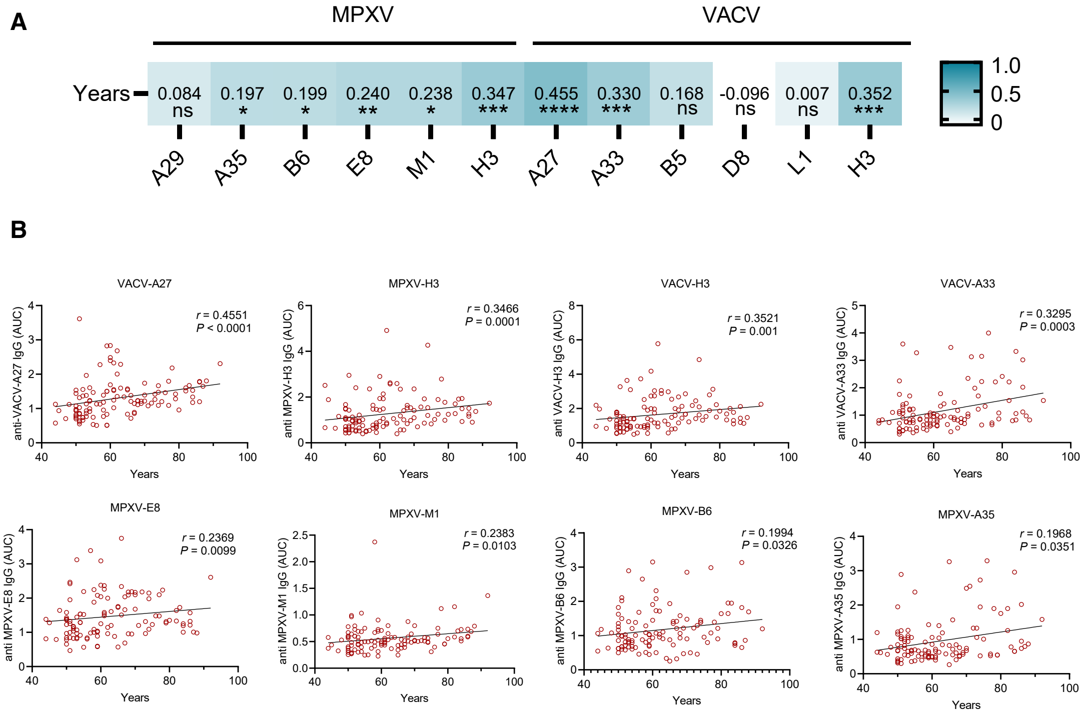

Figure 2. Correlation analysis of age and plasma reactivity in VACV-vaccinated individuals

(A) Spearman's correlation coefficients were used to assess relationships between IgG-binding titers to orthopoxvirus antigens and donor age ($n = 115$). Antibody responses against MPXV (A29, A35, M1, B6, E8, and H3) and VACV (A27, A33, L1, B5, D8, and H3) antigens were compared across age cohorts. The heatmap color scale indicates the strength of correlation between variables.

(B) Scatterplots of age versus antigen-specific IgG binding (ELISA AUC) in historically VACV-vaccinated donors ($n = 115$); each point represents one individual. Spearman's correlation coefficients and p values are reported for each antigen. Representative age-binding correlation plots for selected antigens are shown.

research with larger cohorts and a more detailed analysis of the immune memory associated with previous OPXV exposure is required to determine any potential effects on the MPXV antibody response.

Influence of age and gender on VACV- and MPXV-specific antibody responses

To evaluate the demographic factors associated with OPXV-specific antibody responses, participants enrolled in the study were divided into groups according to age and gender. In detail, the smallpox-vaccinated donors were further divided into five groups in 10-year increments according to their year of birth. VACV and MPXV main surface proteins were used as antigens in ELISA assay. The distribution of antibody titers among participants is shown in Figures 2 and S4. We observe a moderate correlation between VACV-specific IgG area under the curve (AUC) value and age within the VACV-vaccinated individuals. When age groups were compared in 10-year increments, certain group pairs showed statistically significant differences in binding antibody titers. Notably, vaccinated individuals exhibited distinct age-related differences in antibody responses to MPXV antigens. Older individuals generally had higher IgG antibody levels against both VACV and MPXV, which may reflect cumulative effects from multiple historical smallpox vaccinations or repeated exposures to related OPXVs. Although previous studies suggest that antibody levels plateau after two vaccine doses,[20] the continued increase observed with age may indicate variability in vaccination history or immune longevity. In contrast, younger individuals displayed lower antibody titers, likely due to limited or no prior exposure to OPXVs.

To explore the relationship between VACV/MPXV-specific antibodies and gender, we conducted a comparative analysis of antibody responses in male and female participants. Previous study suggests that females may exhibit higher T cell immune responses against both VACV and MPXV compared to males.[21] However, our results showed that specific IgG antibody levels are less consistently differentiated by gender, and binding antibodies show no correlation with gender in VACV-vaccinated individuals (Figure S5).

High nAb response rates, but with low titers, were observed against orthopoxvirus in mpox cases and VACV-vaccinated individuals

We established a focus reduction neutralization test (FRNT) to quantify nAbs against both VACV and MPXV. Four cohorts were evaluated: unvaccinated individuals, VACV-vaccinated individuals, MPXV patients, and convalescents. Neutralization titers for each cohort, along with the proportion exhibiting detectable neutralizing activity, are shown in Figures 3A and 3B. Various levels of OPXV nAbs were detected after infection and historic VACV vaccination. In detail, the baseline neutralization levels of sera from unvaccinated donors were below the critical threshold.

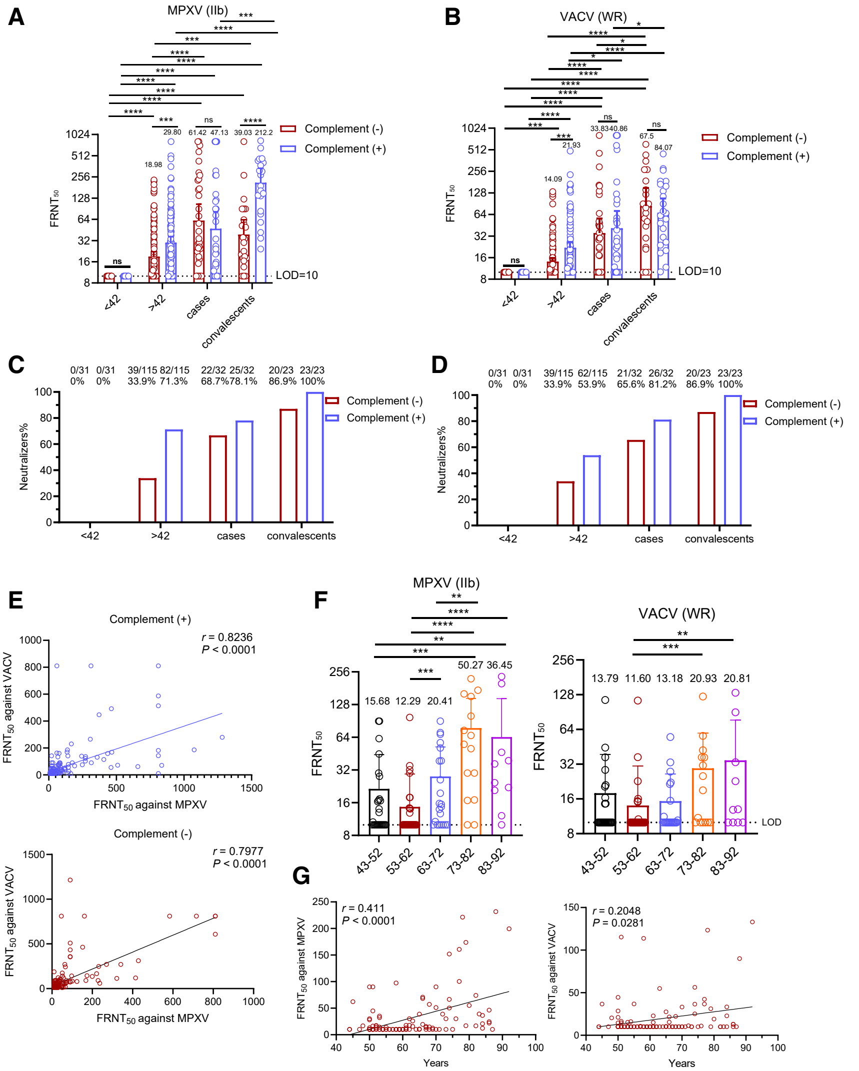

Figure 3. Neutralizing antibodies and factors influencing the antibody response against orthopoxvirus in MPXV-infected and VACV-vaccinated individuals

(A and B) Neutralization titers of plasma against MPXV clade IIb and VACV WR were measured by FRNT with or without 10% guinea pig serum as a complement source. Mean titers are shown above each bar. Dotted lines indicate the limit of detection (LOD = 10). Four cohorts were included: unvaccinated (<42 years, n = 31), VACV vaccinated (>42 years, n = 115), mpox cases (n = 32), and convalescents (n = 23).

(C and D) Seropositivity rates for neutralizing antibodies against MPXV clade IIb (C) and VACV WR (D). Titers below the initial dilution (1:10) were considered negative. Percent positivity for each group is displayed above the bars, with complement-negative conditions in red and complement-positive conditions in blue.

(E) Correlation between MPXV clade IIb and VACV WR neutralizing antibody titers measured with and without complement. Data include all participants (mpox-infected patients, convalescents, historically VACV-vaccinated individuals, and unvaccinated donors). Complement-negative conditions are shown in red, complement-positive in blue.

(F) Evaluation of orthopoxvirus-specific neutralizing antibody titers in historically VACV-vaccinated individuals across age groups (n = 115), measured without complement. The mean titers are displayed above the bars.

(G) The correlation between age and neutralizing titers against MPXV (left) and VACV (right) in historic VACV-vaccinated subjects (n = 115).

y axes in (A, B, and F) are plotted on a $\log_2$ scale. Spearman's correlation coefficients and p values are indicated in (E) and (G). Statistical significance was assessed by one-way ANOVA with multiple comparisons in (A, B, and F). The data comprise 3 independent biological replicates, with datasets including 2–3 technical replicates. Error bars represent mean ± SD; p values are displayed as ns for $p > 0.05$, *$p < 0.05$, **$p < 0.01$ and ***$p < 0.001$.

Plasma samples from VACV-vaccinated individuals exhibited higher nAb titer against OPXV, including MPXV and VACV, than those from unvaccinated control, highlighting the impact of historic VACV vaccination on humoral immunity. Notably, convalescent individuals showed uniformly positive neutralizing responses, with consistently high nAb titers against MPXV, indicating robust antibody-mediated immunity following natural infection.

OPXV demonstrated minimal sensitivity to neutralization. However, the addition of complement improved the detection of responsive individuals and enhanced nAb titer.[22] Among MPXV convalescents, 87% (20/23) and 100% (23/23) were responders in MPXV neutralization tests (NTs) without and with complement, respectively (Figure 3C). For individuals vaccinated with VACV, 33.9% (39/115) and 53.9% (62/115) were responders in VACV NT assays without and with complement, respectively (Figure 3D). The percentage of responders in both VACV-vaccinated individuals and MPXV cases was approximately 1.2- to 2-fold higher in the presence of complement. Furthermore, the addition of complement increased neutralization titers against MPXV in VACV-vaccinated participants, with the median rising from 18.98 to 29.8. However, no significant difference in neutralization titer medium was observed in the sera from MPXV-infected patients, regardless of the presence or absence of complement. Overall, most vaccinated and infected individuals possessed nAbs against MPXV and VACV; however, neutralizing titers were generally low, with median $FRNT_{50}$ values below 61. Low levels of nAbs may hinder their ability to effectively defend against MPXV.

Direct comparison of VACV and MPXV nAb titers revealed a significant positive correlation, both in the presence and absence of complement (Figure 3E). Spearman correlation analysis demonstrated a high correlation coefficient, reflecting their shared antigens and supporting the use of VACV-based vaccines as a model for OPXV. To further investigate the relationship between age and vaccination status, individuals with historic vaccinations were further divided into five groups, each spanning a 10-year age interval. Higher nAb titers (against both MPXV and VACV) were observed in individuals born before

Cell Reports Medicine
Article

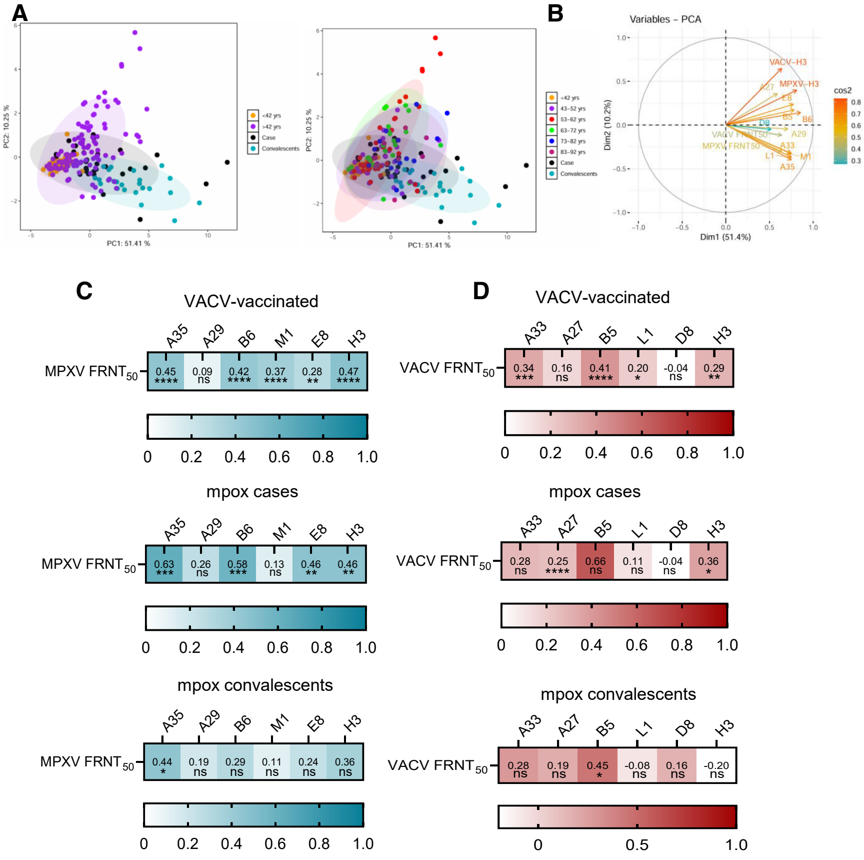

Figure 4. Relationship between binding and neutralizing antibody responses to orthopoxvirus antigens

(A) PCA of antibody binding to six MPXV and six VACV recombinant antigens, together with neutralizing titers against MPXV and VACV. Data are plotted by group, with each colored circle representing an individual sample. Four cohorts were included: unvaccinated (<42 years, n = 31), VACV vaccinated (>42 years, n = 115), mpox cases (n = 32), and convalescents (n = 23).

(B) PCA biplot highlighting the antigen- and neutralization-associated variables contributing most strongly to group separation. Arrow length reflects each variable's contribution, and arrow direction indicates its association with the principal components that separate cohorts.

(C) Correlation between MPXV-specific IgG binding (AUC) and neutralizing titers against MPXV clade IIb across groups with different immunological backgrounds.

(D) Correlation between VACV-specific IgG binding and neutralizing titers against VACV WR strain across heterogeneous immune backgrounds. Spearman's correlation coefficients and p values are reported. The color scale in (C) and (D) represents the strength of correlation between variables.

1950 (Figure 3F). As shown in Figure 3G, a moderate correlation was observed between OPXV neutralizing titer and age, with r = 0.411 (p < 0.0001) for MPXV and r = 0.2048 (p = 0.0281) for VACV.

Immune profiling and antigen correlates of neutralization across distinct orthopoxvirus exposure histories revealed by PCA

Principal-component analysis (PCA) was applied to antibody binding and neutralizing titers against MPXV and VACV antigens to visualize patterns of variation in immune profiles among unvaccinated individuals, VACV-vaccinated individuals, MPXV-infected patients, and convalescents. Unvaccinated individuals exhibited highly similar immune profiles, appearing in close proximity within the PCA space. In contrast, VACV-vaccinated individuals displayed more heterogeneous patterns, which were distinguishable from unvaccinated individuals but partially overlapped with both the unvaccinated group and mpox cases (Figure 4A). mpox cases and convalescents occupied a distinct region of the PCA plot, separate from both unvaccinated and vaccinated individuals, although some overlap with the VACV-vaccinated group was observed. A PCA biplot was used to identify the variables contributing most strongly to the separation between groups (Figure 4B). A35- and M1-targeted antibodies, along with MPXV $FRNT_{50}$ values, contributed most significantly to the separation of the mpox case and convalescents group. In contrast, VACV H3 was most strongly associated with the profiles of the VACV-vaccinated group.

Additionally, we investigated the relationship between binding and nAb responses to OPXV antigens across individuals with distinct immune backgrounds (Figures 4C and 4D). A strong and statistically significant correlation was observed between binding and nAb titers in MPXV-infected individuals. Among the antigens assessed, A35 exhibited the strongest correlation with serum-neutralizing activity, followed by B6 and E8/H3. While the correlation was less pronounced in VACV vaccine recipients, it remained statistically significant.

Quantification of nAb responses targeting multiple MPXV and VACV antigens

Serum nAbs play a pivotal role in conferring protection against OPXV infections. To dissect the antigenic specificity of nAb responses against OPXV surface proteins, we performed a systematic antibody depletion assay targeting multiple viral antigens. In this approach, a volume of serum containing one extracellular enveloped virion (EEV)- or intracellular mature virion (IMV)-specific $PRNT_{50}$ was incubated with specific antigens for 1 h to selectively remove antigen-specific antibodies. The remaining neutralizing activity in the depleted serum was then quantified and compared to that of untreated controls (Figures S6 and S7). We first assessed the contribution of four surface antigens, H3, E8, M1, and A29, to the neutralizing response against the IMV. A heatmap depicting the reduction in neutralizing titers following antigen-specific depletion revealed notable inter-individual variability in antigenic targeting among both mpox-infected individuals and VACV-vaccinated individuals.

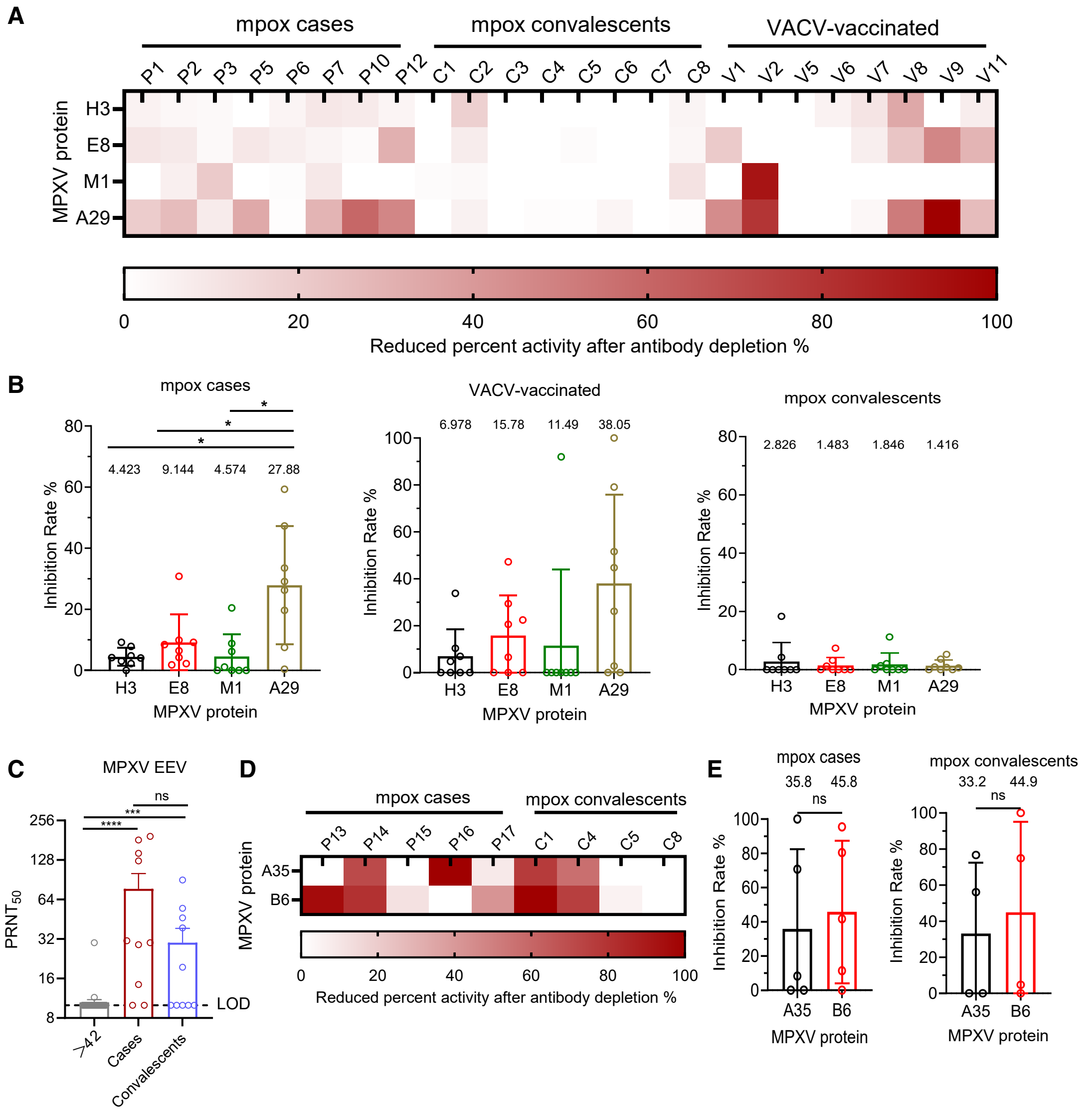

Figure 5. Quantification of neutralizing antibody responses to MPXV antigens by antibody depletion assays

(A) Heatmap showing the percentage reduction in IMV-neutralizing activity after antibody depletion with MPXV antigens (H3, E8, M1, and A29) in plasma from VACV-vaccinated individuals (>42 years, *n* = 8), mpox cases (*n* = 8), and convalescents (*n* = 8). Donor IDs are indicated above each column. Color scale represents the percentage of reduced neutralizing activity against MPXV IMV.

(B) Comparison of reduced IMV-neutralizing activity following incubation with individual MPXV antigens. Mean inhibition ratios are shown above each bar.

(C) Neutralizing titers ($PRNT_{50}$) against MPXV EEV in plasma from mpox cases (*n* = 10), convalescents (*n* = 10), and VACV-vaccinated donors (*n* = 40).

(D) Heatmap showing the percentage reduction in EEV-neutralizing activity after antibody depletion with MPXV antigens (A35, B6). Donor IDs are indicated above each column. Color scale represents the percentage of reduced neutralizing activity against MPXV EEV. Plasma samples were obtained from mpox cases (*n* = 5) and convalescents (*n* = 4).

(E) Comparison of reduced EEV-neutralizing activity following incubation with individual MPXV antigens. Mean inhibition ratios are shown above each bar. Plasma samples from mpox cases (*n* = 5) and convalescents (*n* = 4) were analyzed.

Statistical analysis: two-group comparisons were performed using the Mann-Whitney U test in (E). For multi-group comparisons, one-way ANOVA with multiple comparisons tests was applied in (B) and (C). The data comprise 3 independent biological replicates, with datasets including 2–3 technical replicates. Error bars represent mean ± SD, *p* values are displayed as ns for $p > 0.05$, *$p < 0.05$, ***$p < 0.001$, and ****$p < 0.0001$.

Notably, antibodies targeting A29, E8, and M1 were the major contributors to IMV neutralization. Among these, A29 exerted the most pronounced effect, followed by M1 and E8, suggesting a dominant role in mediating IMV-specific neutralization (Figures 5A and 5B). Next, we examined the nAb response against the EEV, focusing on two key surface antigens, B6 and A35. Neutralizing titers against EEV were first determined (Figure 5C), followed by antigen-specific antibody depletion to assess the relative contribution of each antigen. As shown in the corresponding heatmap, depletion of either B6- or A35-specific antibodies

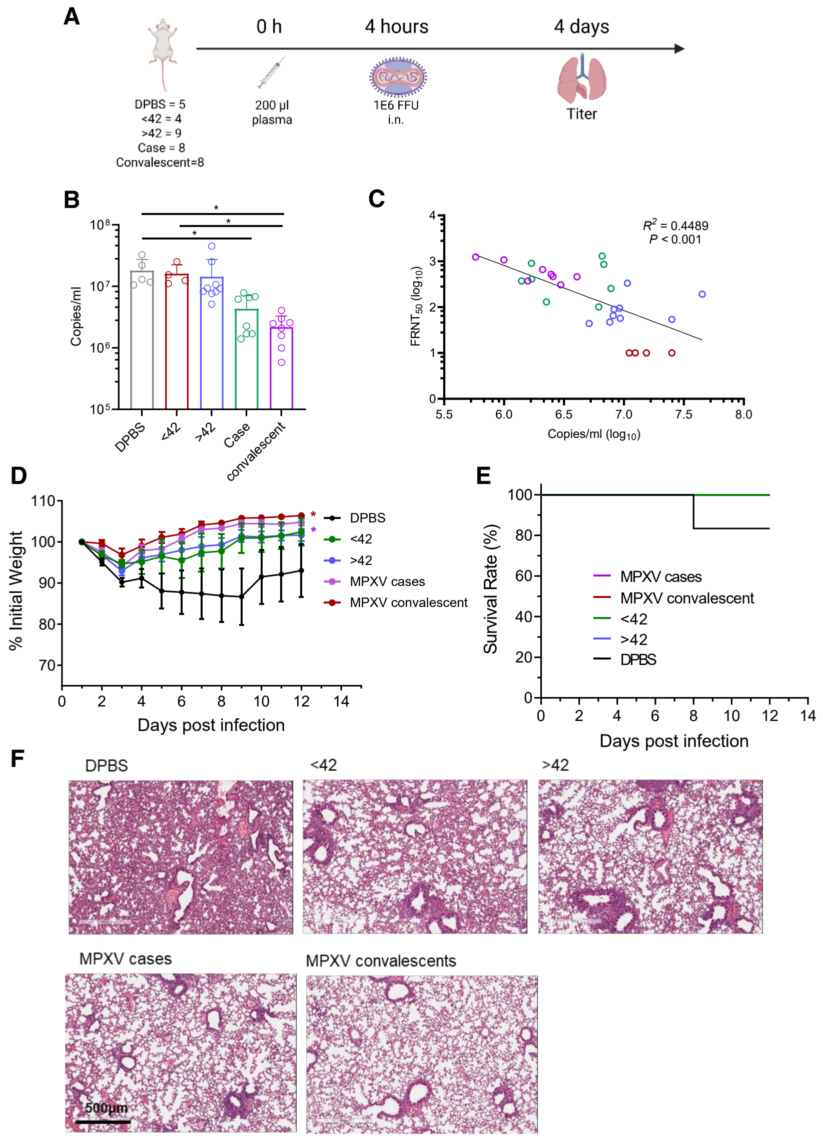

Figure 6. Passive transfer of MPXV-immune plasma-suppressed viral replication *in vivo*

(A) Experimental timeline of plasma adoptive transfer and MPXV clade IIb challenge. BALB/c mice were intravenously injected with 200 µL of plasma from mpox cases (n = 8), convalescents (n = 8), or historically VACV-vaccinated individuals (>42 years, n = 9). Control groups received 200 µL DPBS or plasma from unvaccinated donors (<42 years, n = 4). Mice were challenged with MPXV clade IIb 4 h post-transfer. The schematic was created with BioRender.com.

(B) Viral loads in lung homogenates collected at 4 dpi were quantified by digital PCR and expressed as copies/mL. Each human plasma donor was transferred into a single recipient mouse (one donor to one recipient). Statistical significance was assessed using one-way ANOVA with multiple comparisons tests. The data comprise 3 independent biological replicates, with datasets including 2–3 technical replicates. Error bars represent mean ± SD, p values are displayed as ns for $p > 0.05$, $^*p < 0.05$.

(C) Correlation between $FRNT_{50}$ values of human plasma and lung viral titers in recipient mice. Spearman's correlation coefficients and p values are shown. The analysis included mpox cases (green), convalescents (purple), historically VACV-vaccinated individuals (blue), and VACV-unvaccinated individuals (red). The DPBS control group was excluded. For VACV-unvaccinated donors, all neutralization titers were below the detection limit (LOD = 10) and were therefore set to 10. Color coding matches the samples shown in (B).

(D) Body weight changes were monitored for 12 days (n = 6 mice per group). For survival, weight monitoring, and histopathology analyses, plasma from donors within the same group was pooled prior to transfer to ensure experimental reproducibility and stability.

(E) Survival curves of mice treated with immune plasma following challenge with authentic MPXV clade IIb.

(F) Representative lung histopathology from plasma-treated BALB/c mice collected at 4 dpi. Lungs were fixed in formalin, embedded in paraffin, sectioned, and stained with H&E. DPBS was used as a negative control. Scale bar, 500 µm.

resulted in marked reductions in neutralizing titers, demonstrating that antibodies targeting both antigens play a significant role in mediating EEV neutralization (Figures 5D and 5E).

To further investigate the dominance of VACV-homologous antigens as key targets of the OPXV nAb response, we assessed the relative contribution of three VACV surface antigens, H3, L1, and A27, to VACV-specific neutralization in both mpox patients and individuals with prior VACV vaccination. Among these, L1 elicited robust and durable nAb responses against VACV (Figure S8). Notably, distinct response patterns were observed in individuals with a history of VACV vaccination, suggesting heterogeneity in antigen-specific immunity within this cohort.

Orthopoxvirus immune plasma reduces viral replication in MPXV-infected BALB/c mice

To investigate whether OPXV immune plasma protects against homologous MPXV infection, adoptive transfer experiments were performed in BALB/c mice. Recipient animals were challenged with live MPXV clade IIb 4 hours after plasma transfer (Figure 6A). For lung viral load quantification and correlation analyses, plasma from individual donors was administered to single mice (one donor to one recipient), thereby preserving inter-individual variability. Mice receiving Dulbecco's phosphate-buffered saline (DPBS) or plasma from unvaccinated individuals (<42 years old) showed no protection, whereas transfer of plasma from mpox patients or convalescents significantly reduced lung viral titers at 4 days post infection (dpi) (Figure 6B). These results demonstrate that antibodies elicited during natural infection limit viral replication *in vivo*. Furthermore, nAb levels measured *in vitro* correlated with protective efficacy: $FRNT_{50}$ titers of donor plasma inversely associated with viral loads in recipient lungs (Figure 6C), confirming the functional relevance of nAbs for viral clearance.

For body weight monitoring, survival analysis, and histopathology (Figures 6D–6F), plasma from donors within the same group was pooled prior to transfer (Table S2) to ensure reproducibility and minimize variability. Mice receiving pooled plasma from mpox patients or convalescents exhibited significantly less weight loss and improved survival compared to DPBS controls (Figures 6D and 6E). Histopathological examination further revealed that transfer of convalescent plasma mitigated lung tissue damage at 4 dpi (Figure 6F). Together, these findings indicate that antibodies induced by MPXV infection confer *in vivo* protection, highlighting their potential role as correlates of protection against MPXV.

DISCUSSION

Mpox continues to expand beyond historically endemic regions and sustain human-to-human transmission, underscoring the need to define immune correlates that can guide vaccination and therapeutic strategies.[23,24] In this study, we integrated antigen-resolved serology, neutralization assays, and *in vivo* passive-transfer experiments to characterize humoral immunity to MPXV across heterogeneous exposure histories (historic VACV vaccination, acute infection, and convalescence).

Despite high seropositivity for nAbs after either infection or historic VACV vaccination, the absolute titers were generally modest, consistent with reports that third-generation MVA-BN (JYNNEOS/IMVANEX) elicits limited cross-neutralization against MPXV.[25] Our antigen-level analyses converge with recent antigen-discovery work showing that a subset of IMV and EEV surface proteins disproportionately shape functional humoral immunity: A29, M1, and E8 on IMV and A35 and B6 on EEV emerged as dominant nAb targets across exposure histories,[26–29] These observations are concordant with multivalent vaccine studies in mice and non-human primates demonstrating that inclusion of both EEV and IMV antigens broadens and strengthens protection.[30,31]

Population factors also modulated antibody magnitude. Among historically VACV-vaccinated donors, binding and neutralization increased with age, plausibly reflecting dose histories and longevity of orthopox immunity,[32] while we observed no robust gender-associated differences in binding responses. These trends align with meta-analytic evidence linking antibody levels to estimated vaccine effectiveness and with durability analyses suggesting that boosting schedules influence long-term protection.[33] Notably, pre-existing VACV immunity did not blunt induction of MPXV-specific responses in acute cases in our cohort. This may be attributed to the long interval since vaccination, exceeding 40 years, and the relatively low residual antibody levels.

Beyond the six antigens profiled here (IMV: A29, and M1, E8; EEV: A35, B6, and H3), recent work expands protective membrane targets and links antigen-specific antibodies to *in vivo* protection. Human anti-A35 mAbs, focused on a conserved site, reduce viremia, tissue viral loads, and lesions in mice and macaques, establishing A35 as a dominant EEV epitope and therapeutic target.[34,35] An antigen-agnostic screen also identified OPG153 (A28) as a conserved, broadly neutralizing target[36]; OPG153 immunization elicited stronger serum neutralization than MVA-BN. In addition, A16/G9 from the entry-fusion complex mediates cross-neutralization and vaccine protection.[37] These advances align with multivalent vaccine strategies and multivalent vaccine design. Combining OPG153 and A16/G9 with A29/M1/E8 and A35/B6/H3, prioritizing conserved, structurally validated epitopes, merits evaluation to determine whether next-generation cocktails can increase neutralization breadth and potency.

By antibody-depletion mapping, we quantified antigenic contributions to neutralization at the serum level. For IMV, A29 exerted the largest impact, followed by M1 and E8; for EEV, both A35 and B6 were necessary for maximal activity, echoing recent human monoclonal antibody studies that identified non-competing B6-directed nAbs with *in vivo* efficacy.[38] Functionally, passive transfer of immune plasma from mpox patients and convalescents reduced lung viral burden and ameliorated disease in mice, and $FRNT_{50}$ values inversely tracked pulmonary virus loads, directly linking *in vitro* neutralization to *in vivo* control in this model.

This work advances the field in three ways. First, it provides a head-to-head, antigen-resolved map of MPXV nAb targets spanning IMV and EEV in both infection and vaccination contexts. Second, it integrates population variables (age, prior vaccination) with functional readouts to refine correlates of protection beyond total IgG. Third, it functionally validates human antibody repertoires by *in vivo* plasma transfer, helping to bridge *in vitro* neutralization and outcome.

Limitations of the study

This work has several limitations. First, although recent outbreaks have involved both clade IIb (2022–2023) and clade Ib (2023–2024), all live-virus experiments here used a clade IIb clinical isolate; cross-clade analyses (I/Ib/IIb) are needed to test generalizability. Second, historical smallpox vaccine exposures were heterogeneous worldwide (e.g., Tian Tan, Lister, Wyeth), and our cohort did not include recipients of third-generation MVA-BN (JYNNEOS); region- and platform-specific studies are warranted. Third, human sampling was cross-sectional, largely mild disease, and drawn from a single geographic area, limiting inference on kinetics and severity strata. Finally, the intranasal mouse challenge model may not fully recapitulate predominant human transmission routes; evaluating additional exposure routes (e.g., intraperitoneal, intravenous, and contact) and dose-timing regimens, and incorporating cellular and mucosal immunity readouts, will better define protective thresholds and mechanisms.

RESOURCE AVAILABILITY

Lead contact

Further information and requests for resources, and data should be directed to and will be fulfilled by the lead contact, Jincun Zhao (zhaojincun@gird.cn).

Materials availability

This study did not generate any new unique reagents.

Data and code availability

- All data reported in this paper will be shared by the lead contact upon request.
- This paper does not report any original code.
- Any additional information required to reanalyze the data reported in this paper is available from the lead contact upon request.

ACKNOWLEDGMENTS

This project was supported by grants from the National Key Research and Development Program of China (2024YFA0920001 to Y.W.), the National Natural Science Foundation of China (82572034 to Y.W. and 82025001, 82495200, and 82495203 to J.Z.), Guangzhou National Laboratory and State Key Laboratory of Respiratory Disease (GZNL2024B01001 to Y.W.), Guangdong Basic and Applied Research Projects (2023B1515020040 to Y.W.), Science and Technology Planning Project of Guangzhou City (2024A03J1230 to J. Zhao, 2025B04J0006 to L. Zhang, and 2023A04J1259 to L. Zhou), Major Demonstration Project for Scientific and Technological Innovation of Inner Mongolia Autonomous Region (2025ZDSF0021), Key Research and Development and Achievement Transformation Project (2025YFDZ0145), the Key Project of Medicine Discipline of Guangzhou (2025–2027-11), and the Science and Technology Project of General Administration of Customs, P.R. China (2025HK221 to L. Zhang). We thank the Biobank for Respiratory Disease in the National Clinical Research Center for Respiratory Disease (BRD-NCRCRD, Guangzhou, Southern China).

AUTHOR CONTRIBUTIONS

Jincun Zhao, P.Q., J.D., Jingxian Zhao, and Y.W. initiated and coordinated the project. Y.W., L. Zhang, L. Zhou, J.C., and Z.Z evaluated the neutralizing activity and protective experiments against MPXV *in vivo*. T.W., P.W., A.Z., R.C., J. Zhang, and Z.L. performed ELISA detection. C.C., Y.Z., and Q.Z. analyzed the data. J.S. and Y.S. provided VACV WR strain and technical support. Y.W. wrote and revised the manuscript. All authors read and approved the final version of the manuscript.

DECLARATION OF INTERESTS

The authors declare no conflicts of interest.

STAR★METHODS

Detailed methods are provided in the online version of this paper and include the following:

- KEY RESOURCES TABLE
- EXPERIMENTAL MODEL AND STUDY PARTICIPANT DETAILS
  - Human subjects
  - Animal care and use
  - Viruses and cell lines
- METHOD DETAILS
  - Procedures
  - Detection of MPXV- and VACV-specific IgG antibody by single antigen ELISA
  - Preparation of MPXV viral stocks for neutralization assays
  - Detection of MPXV- and VACV-specific neutralization antibodies by FRNT
  - Detection of MPXV-specific neutralization antibodies by PRNT
  - Antibody depletion experiments
  - Immune plasma treatment and MPXV infection of mice
  - MPXV DNA detection by digital PCR
- QUANTIFICATION AND STATISTICAL ANALYSIS

SUPPLEMENTAL INFORMATION

Supplemental information can be found online at https://doi.org/10.1016/j.xcrm.2025.102483.

Received: December 14, 2024
Revised: September 16, 2025
Accepted: November 11, 2025
Published: December 8, 2025

REFERENCES

1. Titanji, B.K., Hazra, A., and Zucker, J. (2024). Mpox Clinical Presentation, Diagnostic Approaches, and Treatment Strategies: A Review. JAMA *332*, 1652–1662. https://doi.org/10.1001/jama.2024.21091.
2. Sarker, R., Roknuzzaman, A.S.M., Shahriar, M., Bhuiyan, M.A., and Islam, M.R. (2023). The WHO has ended public health emergency of international concern for mpox: assessment of upside and downside of this decision. Int. J. Surg. *109*, 3238–3239. https://doi.org/10.1097/js9.0000000000000545.
3. Lum, F.M., Torres-Ruesta, A., Tay, M.Z., Lin, R.T.P., Lye, D.C., Rénia, L., and Ng, L.F.P. (2022). Monkeypox: disease epidemiology, host immunity and clinical interventions. Nat. Rev. Immunol. *22*, 597–613. https://doi.org/10.1038/s41577-022-00775-4.
4. Ahmed, S.F., Sohail, M.S., Quadeer, A.A., and McKay, M.R. (2022). Vaccinia-Virus-Based Vaccines Are Expected to Elicit Highly Cross-Reactive Immunity to the 2022 Monkeypox Virus. Viruses *14*, 1960. https://doi.org/10.3390/v14091960.
5. Altenburg, A.F., van Trierum, S.E., de Bruin, E., de Meulder, D., van de Sandt, C.E., van der Klis, F.R.M., Fouchier, R.A.M., Koopmans, M.P.G., Rimmelzwaan, G.F., and de Vries, R.D. (2018). Effects of pre-existing orthopoxvirus-specific immunity on the performance of Modified Vaccinia virus Ankara-based influenza vaccines. Sci. Rep. *8*, 6474. https://doi.org/10.1038/s41598-018-24820-2.
6. Gilchuk, I., Gilchuk, P., Sapparapu, G., Lampley, R., Singh, V., Kose, N., Blum, D.L., Hughes, L.J., Satheshkumar, P.S., Townsend, M.B., et al. (2016). Cross-Neutralizing and Protective Human Antibody Specificities to Poxvirus Infections. Cell *167*, 684–694.e9. https://doi.org/10.1016/j.cell.2016.09.049.
7. McDermott, A. (2021). Core Concept: Herd immunity is an important-and often misunderstood-public health phenomenon. Proc. Natl. Acad. Sci. USA *118*, e2107692118. https://doi.org/10.1073/pnas.2107692118.
8. Nguyen, P.Y., Ajisegiri, W.S., Costantino, V., Chughtai, A.A., and MacIntyre, C.R. (2021). Reemergence of Human Monkeypox and Declining Population Immunity in the Context of Urbanization, Nigeria, 2017-2020. Emerg. Infect. Dis. *27*, 1007–1014. https://doi.org/10.3201/eid2704.203569.
9. Simpson, K., Heymann, D., Brown, C.S., Edmunds, W.J., Elsgaard, J., Fine, P., Hochrein, H., Hoff, N.A., Green, A., Ihekweazu, C., et al. (2020). Human monkeypox - After 40 years, an unintended consequence of smallpox eradication. Vaccine *38*, 5077–5081. https://doi.org/10.1016/j.vaccine.2020.04.062.
10. Thornhill, J.P., Barkati, S., Walmsley, S., Rockstroh, J., Antinori, A., Harrison, L.B., Palich, R., Nori, A., Reeves, I., Habibi, M.S., et al. (2022). Monkeypox Virus Infection in Humans across 16 Countries - April-June 2022. N. Engl. J. Med. *387*, 679–691. https://doi.org/10.1056/NEJMoa2207323.
11. Ghazy, R.M., Elrewany, E., Gebreal, A., ElMakhzangy, R., Fadl, N., Elbanna, E.H., Tolba, M.M., Hammad, E.M., Youssef, N., Abosheaishaa, H., et al. (2023). Systematic Review on the Efficacy. Vaccines (Basel). *11*, 1708. https://doi.org/10.3390/vaccines11111708.
12. Jia, H., Sha, T., Zhao, S., Su, W., Liu, P., Zhen, R., Li, P., Zhou, L., Xu, Y., Wen, Y., et al. (2023). Genomic and epidemiological perspectives on the first local sporadic cases of Mpox in China. Emerg. Microbes Infect. *12*, 2245932. https://doi.org/10.1080/22221751.2023.2245932.
13. Mariotti, S., Venturi, G., Chiantore, M.V., Teloni, R., De Santis, R., Amendola, A., Fortuna, C., Marsili, G., Grilli, G., Lia, M.S., et al. (2024). Antibodies Induced by Smallpox Vaccination after at Least 45 Years Cross-React with and In Vitro Neutralize Mpox Virus: A Role for Polyclonal B Cell Activation? Viruses *16*, 620. https://doi.org/10.3390/v16040620.
14. Christodoulidou, M.M., and Mabbott, N.A. (2023). Efficacy of smallpox vaccines against Mpox infections in humans. Immunother. Adv. *3*, ltad020. https://doi.org/10.1093/immadv/ltad020.
15. Otter, A.D., Jones, S., Hicks, B., Bailey, D., Callaby, H., Houlihan, C., Rampling, T., Gordon, N.C., Selman, H., Satheshkumar, P.S., et al. (2023).

CellPress
OPEN ACCESS

Monkeypox virus-infected individuals mount comparable humoral immune responses as Smallpox-vaccinated individuals. Nat. Commun. *14*, 5948. https://doi.org/10.1038/s41467-023-41587-x.

16. Manenti, A., Solfanelli, N., Cantaloni, P., Mazzini, L., Leonardi, M., Benincasa, L., Piccini, G., Marchi, S., Boncioli, M., Spertilli Raffaelli, C., et al. (2023). Evaluation of Monkeypox- and Vaccinia virus-neutralizing antibodies in human serum samples after vaccination and natural infection. Front. Public Health *11*, 1195674. https://doi.org/10.3389/fpubh.2023.1195674.

17. Zaeck, L.M., Lamers, M.M., Verstrepen, B.E., Bestebroer, T.M., van Royen, M.E., Götz, H., Shamier, M.C., van Leeuwen, L.P.M., Schmitz, K.S., Alblas, K., et al. (2023). Low levels of monkeypox virus-neutralizing antibodies after MVA-BN vaccination in healthy individuals. Nat. Med. *29*, 270–278. https://doi.org/10.1038/s41591-022-02090-w.

18. Raccagni, A.R., Mancon, A., Diotallevi, S., Lolatto, R., Bruzzesi, E., Gismondo, M.R., Castagna, A., Mileto, D., and Nozza, S. (2024). Monkeypox Virus Neutralizing Antibodies at Six Months from Mpox Infection: Virologic Factors Associated with Poor Immunologic Response. Viruses *16*, 681. https://doi.org/10.3390/v16050681.

19. Xia, A., Wang, X., He, J., Wu, W., Jiang, W., Xue, S., Zhang, Q., Gao, Y., Han, Y., Li, Y., et al. (2023). Cross-reactive antibody response to Monkeypox virus surface proteins in a small proportion of individuals with and without Chinese smallpox vaccination history. BMC Biol. *21*, 205. https://doi.org/10.1186/s12915-023-01699-8.

20. Hammarlund, E., Lewis, M.W., Hansen, S.G., Strelow, L.I., Nelson, J.A., Sexton, G.J., Hanifin, J.M., and Slifka, M.K. (2003). Duration of antiviral immunity after smallpox vaccination. Nat. Med. *9*, 1131–1137. https://doi.org/10.1038/nm917.

21. Li, M., Guo, Y., Deng, Y., Gao, W., Huang, B., Yao, W., Zhao, Y., Zhang, Q., Huang, M., Liu, M., et al. (2024). Long-lasting humoral and cellular memory immunity to vaccinia virus Tiantan provides pre-existing immunity against mpox virus in Chinese population. Cell Rep. *43*, 113609. https://doi.org/10.1016/j.celrep.2023.113609.

22. Hubert, M., Guivel-Benhassine, F., Bruel, T., Porrot, F., Planas, D., Vanhomwegen, J., Wiedemann, A., Burrel, S., Marot, S., Palich, R., et al. (2023). Complement-dependent mpox-virus-neutralizing antibodies in infected and vaccinated individuals. Cell Host Microbe *31*, 937–948.e4. https://doi.org/10.1016/j.chom.2023.05.001.

23. Lee, S.S., Traore, T., and Zumla, A. (2024). The WHO mpox public health emergency of international concern declaration: Need for reprioritisation of global public health responses to combat the MPXV Clade I epidemic. Int. J. Infect. Dis. *147*, 107227. https://doi.org/10.1016/j.ijid.2024.107227.

24. Du, M., Liu, M., Niu, B., and Liu, J. (2024). The global alarm bell is ringing due to the threat of potential severe cases and deaths caused by clade I of monkeypox virus. Lancet Infect. Dis. *24*, e616–e617. https://doi.org/10.1016/s1473-3099(24)00530-9.

25. Jandrasits, D., Züst, R., Siegrist, D., Engler, O.B., Weber, B., Schmidt, K.M., and Jonsdottir, H.R. (2024). Third-generation smallpox vaccines induce low-level cross-protecting neutralizing antibodies against Monkeypox virus in laboratory workers. Heliyon *10*, e31490. https://doi.org/10.1016/j.heliyon.2024.e31490.

26. Sang, Y., Zhang, Z., Liu, F., Lu, H., Yu, C., Sun, H., Long, J., Cao, Y., Mai, J., Miao, Y., et al. (2023). Monkeypox virus quadrivalent mRNA vaccine induces immune response and protects against vaccinia virus. Signal Transduct. Target. Ther. *8*, 172. https://doi.org/10.1038/s41392-023-01432-5.

27. Su, C., Li, S., Wen, Y., Geng, X., Yin, Q., Wang, Y., Xiong, Y., and Liu, Z. (2024). A Quadrivalent mRNA Immunization Elicits Potent Immune Responses against Multiple Orthopoxviral Antigens and Neutralization of Monkeypox Virus in Rodent Models. Vaccines *12*, 385. https://doi.org/10.3390/vaccines12040385.

28. Freyn, A.W., Atyeo, C., Earl, P.L., Americo, J.L., Chuang, G.Y., Natarajan, H., Frey, T.R., Gall, J.G., Moliva, J.I., Hunegnaw, R., et al. (2023). An mpox virus mRNA-lipid nanoparticle vaccine confers protection against lethal orthopoxviral challenge. Sci. Transl. Med. *15*, eadg3540. https://doi.org/10.1126/scitranslmed.adg3540.

29. Hou, F., Zhang, Y., Liu, X., Murad, Y.M., Xu, J., Yu, Z., Hua, X., Song, Y., Ding, J., Huang, H., et al. (2023). mRNA vaccines encoding fusion proteins of monkeypox virus antigens protect mice from vaccinia virus challenge. Nat. Commun. *14*, 5925. https://doi.org/10.1038/s41467-023-41628-5.

30. Zhang, R.R., Wang, Z.J., Zhu, Y.L., Tang, W., Zhou, C., Zhao, S.Q., Wu, M., Ming, T., Deng, Y.Q., Chen, Q., et al. (2023). Rational development of multicomponent mRNA vaccine candidates against mpox. Emerg. Microbes Infect. *12*, 2192815. https://doi.org/10.1080/22221751.2023.2192815.

31. Ye, Q., Zhang, D., Zhang, R.-R., Xu, Q., Huang, X.-Y., Huang, B., Sun, M.-X., Cong, Z., Zhu, L., Ma, J., et al. (2024). A penta-component mpox mRNA vaccine induces protective immunity in nonhuman primates. Nat. Commun. *15*, 10611. https://doi.org/10.1038/s41467-024-54909-4.

32. Dee, K., Manali, M., Bissett, L.A., Bone, J., Magill, C., Davis, C., Willett, B.J., and Murcia, P.R. (2024). Smallpox vaccination campaigns resulted in age-associated population cross-immunity against monkeypox virus. J. Gen. Virol. *105*, 001999. https://doi.org/10.1099/jgv.0.001999.

33. Berry, M.T., Khan, S.R., Schlub, T.E., Notaras, A., Kunasekaran, M., Grulich, A.E., MacIntyre, C.R., Davenport, M.P., and Khoury, D.S. (2024). Predicting vaccine effectiveness for mpox. Nat. Commun. *15*, 3856. https://doi.org/10.1038/s41467-024-48180-w.

34. Ju, B., Liu, C., Zhang, J., Li, Y., Yang, H., Zhou, B., Huang, B., Ma, J., Lu, J., Cheng, L., et al. (2025). Structurally conserved human anti-A35 antibodies protect mice and macaques from mpox virus infection. Cell *188*, 6253–6265.e14. https://doi.org/10.1016/j.cell.2025.08.005.

35. Fantin, R.F., Yuan, M., Park, S.C., Bozarth, B., Cohn, H., Ignacio, M., Earl, P., Civljak, A., Laghlali, G., Zhang, D., et al. (2025). Human monoclonal antibodies targeting A35 protect from death caused by mpox. Cell *188*, 6236–6252.e18. https://doi.org/10.1016/j.cell.2025.08.004.

36. Paciello, I., Zhou, L., Rundlet, E.J., Realini, G., Perrone, F., Pierleoni, G., Troisi, M., Postal, J., Guivel-Benhassine, F., Porrot, F., et al. (2025). Antigen-agnostic identification of poxvirus broadly neutralizing antibodies targeting OPG153. Preprint at bioRxiv. https://doi.org/10.1101/2025.08.15.670377.

37. Meola, A., Vernuccio, R., Battini, L., Albericio, G., Delgado, P., Bamford, R., Pokorny, L., Broutin, M., Martínez León, A., Gallien, S., et al. (2025). Structural basis of poxvirus fusion regulation and anti-A16/G9 antibody-mediated neutralization and protection. Cell *188*, 6266–6282.e18. https://doi.org/10.1016/j.cell.2025.07.040.

38. Zhao, R., Wu, L., Sun, J., Liu, D., Han, P., Gao, Y., Zhang, Y., Xu, Y., Qu, X., Wang, H., et al. (2024). Two noncompeting human neutralizing antibodies targeting MPXV B6 show protective effects against orthopoxvirus infections. Nat. Commun. *15*, 4660. https://doi.org/10.1038/s41467-024-48312-2.

39. Zuiani, A., Dulberger, C.L., De Silva, N.S., Marquette, M., Lu, Y.J., Palowitch, G.M., Dokic, A., Sanchez-Velazquez, R., Schlatterer, K., Sarkar, S., et al. (2024). A multivalent mRNA monkeypox virus vaccine (BNT166) protects mice and macaques from orthopoxvirus disease. Cell *187*, 1363–1373.e12. https://doi.org/10.1016/j.cell.2024.01.017.

40. Qu, Y., Tai, W., Ma, E., Jiang, Q., Fan, M., Xiao, W., Tian, C., Liu, Y., Liu, J., Wang, X., et al. (2025). Generation and characterization of neutralizing antibodies against M1R and B6R proteins of monkeypox virus. Nat. Commun. *16*, 3100. https://doi.org/10.1038/s41467-025-58180-z.

41. Li, M., Chen, J., Wang, F., Kuang, J., Peng, Y., Asghar, S., Zhao, W., Yang, Y., and Shen, C. (2025). Bispecific antibodies targeting MPXV A29 and B6 demonstrate efficacy against MPXV infection. J. Virol. *99*, e0232024. https://doi.org/10.1128/jvi.02320-24.

42. Esqueda, A., Sun, H., Bonner, J., Lai, H., Jugler, C., Kibler, K.V., Steinkellner, H., and Chen, Q. (2023). A Monoclonal Antibody Produced in Glycoengineered Plants Potently Neutralizes Monkeypox Virus. Vaccines *11*, 1179. https://doi.org/10.3390/vaccines11071179.

43. Pütz, M.M., Midgley, C.M., Law, M., and Smith, G.L. (2006). Quantification of antibody responses against multiple antigens of the two infectious forms of Vaccinia virus provides a benchmark for smallpox vaccination. Nat. Med. *12*, 1310–1315. https://doi.org/10.1038/nm1457.

44. Benhnia, M.R.E.I., McCausland, M.M., Su, H.P., Singh, K., Hoffmann, J., Davies, D.H., Felgner, P.L., Head, S., Sette, A., Garboczi, D.N., and Crotty, S. (2008). Redundancy and plasticity of neutralizing antibody responses are cornerstone attributes of the human immune response to the smallpox vaccine. J. Virol. *82*, 3751–3768. https://doi.org/10.1128/jvi.02244-07.

45. Americo, J.L., Earl, P.L., and Moss, B. (2023). Virulence differences of mpox (monkeypox) virus clades I, IIa, and IIb.1 in a small animal model. Proc. Natl. Acad. Sci. USA *120*, e2220415120. https://doi.org/10.1073/pnas.2220415120.

46. Doty, J.B., Malekani, J.M., Kalemba, L.N., Stanley, W.T., Monroe, B.P., Nakazawa, Y.U., Mauldin, M.R., Bakambana, T.L., Liyandja Dja Liyandja, T., Braden, Z.H., et al. (2017). Assessing Monkeypox Virus Prevalence in Small Mammals at the Human-Animal Interface in the Democratic Republic of the Congo. Viruses *9*, 283. https://doi.org/10.3390/v9100283.

47. Falendysz, E.A., Lopera, J.G., Rocke, T.E., and Osorio, J.E. (2023). Monkeypox Virus in Animals: Current Knowledge of Viral Transmission and Pathogenesis in Wild Animal Reservoirs and Captive Animal Models. Viruses *15*, 905. https://doi.org/10.3390/v15040905.

48. Cheng, L., Huang, W., Duan, M., Li, Z., Chen, Q., Zhang, M., and Zhang, Z. (2024). Pathogenic BALB/c mice infection model for evaluation of mpox countermeasures. Cell Discov. *10*, 105. https://doi.org/10.1038/s41421-024-00739-z.

STAR★METHODS

KEY RESOURCES TABLE

REAGENT or RESOURCE	SOURCE	IDENTIFIER
Antibodies		
Goat anti-Human IgG-Fc HRP-conjugated antibody	Jackson	109-035-088; RRID:AB_2337584
anti-VACV-polyclonal antibodies	Abcam	ab35219; RRID:AB_778768
Bacterial and virus strains		
MPXV clade IIb	This paper	GenBank: PX488348
Vaccinia virus (WR)	ATCC	No. VR-1354
Biological samples		
Plasma samples	This study	N/A
Chemicals, peptides, and recombinant proteins		
MPXV A29 protein	SinoBiological	40891-V08E
MPXV A35 protein	SinoBiological	40886-V08H
MPXV M1 protein	SinoBiological	40904-V07H
MPXV B6 protein	SinoBiological	40902-V08H
MPXV E8 protein	SinoBiological	40890-V08B
MPXV H3 protein	SinoBiological	40893-V08H1
VACV A27 protein	SinoBiological	40897-V07E
VACV A33 protein	SinoBiological	40896-V07E
VACV L1 protein	SinoBiological	40903-V07H
VACV B5 protein	SinoBiological	40900-V08H
VACV D8 protein	SinoBiological	40898-V08H
VACV H3 protein	Abmart	EHV4943
TMB substrate	Biohao Biotechnology	N0160
KPL TrueBlue substrate	Seracare	5510–0030
guinea pig serum	Bersee	BM361Y
0.25%Trypsin-EDTA	Gibco	25200–056
Tween 20	Solarbio	T8220
Critical commercial assays		
Viral DNA/RNA Extraction Kit	Vazyme	RM401-04
mpox Virus Nucleic Acid Detection Kit	TargetingONE	no. 13500
Experimental models: Cell lines		
Vero 81	ATCC	CCL-81
Vero E6 cell	ATCC	CRL-1586
Experimental models: Organisms/strains		
BALB/c mice	Gem Pharmatech	N000020
Software and algorithms		
Prism	GraphPad	V8.0

EXPERIMENTAL MODEL AND STUDY PARTICIPANT DETAILS

Human subjects

A total of 146 human serum samples from non-mpox individuals were selected and stratified by age, including 31 VACV-unvaccinated individuals (<42 years) and 115 individuals with a presumed history of childhood smallpox (VACV) vaccination (>42 years) in China. These samples were anonymously collected in Guangzhou between 2022 and 2024 as residual specimens from unrelated

diagnostic procedures and archived at the First Affiliated Hospital of Guangzhou Medical University. For each sample, only the date of collection, age, and gender of the donor were recorded. In addition, 32 serum samples from laboratory-confirmed MPXV-positive patients (acute phase) were obtained from the Guangzhou Center for Disease Control and Prevention (CDC). Laboratory nucleic acid testing confirmed that all MPXV strains from these infected patients belonged to clade IIb, consistent with previous reports of circulating strains in Guangzhou during 2023–2024.[12] Furthermore, 23 plasma samples from mpox convalescents were collected 6–9 months after infection. This study was approved by the Ethics Committee of the Guangzhou CDC (GZCDC-ECHR-2023P0059), and written informed consent was obtained from all participants. All experiments with authentic MPXV were performed at the Guangzhou Customs District Technology Center BSL-3 Laboratory.

Animal care and use

Female BALB/c mice (6–8 weeks old) were obtained from GemPharmatech Co., Ltd. (Guangdong, China) and acclimated for 2–3 days prior to experimental procedures. On the day of infection, mice were anesthetized via intraperitoneal injection of avertin. Following infection, animals were monitored daily for clinical signs and adverse events, and euthanized at the study endpoint on day 10 post-infection. All procedures involving plasma adoptive transfer and MPXV challenge in mice were approved by the Institutional Animal Care and Use Committee of the First Affiliated Hospital of Guangzhou Medical University (approval no. 2024-0529).

Viruses and cell lines

mpox virus (MPXV) was isolated from a swab of a characteristic skin lesion of an MPXV-positive patient using Vero 81 cells and stored at the biosafety level 3 (BSL-3) laboratory of the Guangzhou Customs District Technology Center. Genomic analysis confirmed that the isolate belonged to lineage IIb (GenBank: PX488348). MPXV was subsequently propagated and titrated in Vero 81 cells. Vero 81 (CCL-81) cells were sourced from the American Type Culture Collection (ATCC, Virginia, USA). The vaccinia virus (VACV) Western Reserve (WR) reference strain (NCBI: NC_006998; ATCC: VR-1354) was obtained from the ATCC and propagated in Vero E6 (ATCC: CCL-37). All cell lines used in this study were routinely tested for mycoplasma and found to be mycoplasma-free.

METHOD DETAILS

Procedures

Clinical information (age, gender, and vaccination history) was collected from all participants. Serological profiling was conducted using in-house ELISAs against MPXV antigens (A29, A35, M1, B6, E8L, H3) and corresponding VACV homologs (A27, A33, L1, B5, D8L, H3). Neutralization activity against MPXV was evaluated by focus reduction (FRNT) and plaque reduction (PRNT) assays. Seropositivity was defined as the mean optical density plus three standard deviations of sera from unvaccinated individuals under 20 years. For adoptive transfer experiments, 6–8-week-old female BALB/c mice received 200 μL of human plasma intravenously, followed by MPXV (clade IIb) challenge after 4 h. Control groups received DPBS or plasma from unvaccinated donors. Lung viral titers were measured by digital PCR. Digital PCR was performed with a commercial detection kit (TargetingONE Corporation, cat. no. 13500) following the manufacturer's protocol.

Detection of MPXV- and VACV-specific IgG antibody by single antigen ELISA

Clinical sample plasma was collected and analyzed for the presence of MPXV- and VACV-specific IgG antibodies to the main surface proteins using indirect ELISA as previously reported.[39] Briefly, ELISA plates were coated with 1 μg/mL of purified recombinant protein, including MPXV A29, A35, M1, B6, E8L, H3, and VACV A27, A33, L1, B5, D8L, H3. The plates were covered and incubated at 4°C overnight. The following morning the plates were washed with PBST and blocked with blocking buffer (10% FBS). 4-fold serial dilutions of human sera, starting from 1:100, were added to the coated plates, which were then incubated for one hour at 37°C. After the washing step, 100 μL/well of Goat anti-Human IgG-Fc HRP-conjugated antibody (Jackson ImmunoResearch, catalog 109-035-088) was added. The plates were incubated at 37°C for 30 min; 100 μL/well of TMB substrate (Biohao Biotechnology, N0160) was added. The plates were then incubated in the dark at room temperature for 10 min at RT. Development was stopped by adding 100 mL 0.2 M H_2SO_4 to each well. Absorbance was read at 450 nm using a plate reader (BioTek). In addition, paraformaldehyde-inactivated vaccinia virus and MPXV were coated with 5000 FFU/well, and used for ELISA assay, respectively.

Preparation of MPXV viral stocks for neutralization assays

Extracellular enveloped virus (EEV) stocks of MPXV were produced using previously described approaches[40,41] with minor adaptations. In brief, Vero 81 cells were infected at a multiplicity of infection (MOI) of 0.2 focus-forming units (FFU) per cell. Cell culture supernatants were collected once extensive cytopathic effect was evident (typically by day 3 for clade IIb MPXV), clarified by centrifugation at 8,000 × g for 15 min, and stored at 4°C for short-term use (≤2 weeks). To minimize contamination with intracellular mature virions (IMV), titration was carried out in the presence of the IMV-specific neutralizing antibody anti-L1R (clone 7D11). For preparation of viral stocks containing both IMV and EEV, Vero E6 cells were infected at an MOI of 0.2 FFU per cell. At 72 h post-infection, supernatants and infected cells were harvested together and subjected to three cycles of freeze–thaw to release

intracellular virions. Following clarification at 8,000 × g for 15 min, the supernatant was aliquoted and stored at −80°C for long-term preservation.

Detection of MPXV- and VACV-specific neutralization antibodies by FRNT

Human sera neutralization activity against MPXV was determined by focus reduction neutralization test (FRNT) based on previous reports.[42] In detail, Vero 81 cells were plated in tissue culture plates 24 h before the assay. Sera were serially diluted in a 96-well microtiter plate using DMEM with 2% heat-inactivated FBS (D2 medium). Meanwhile, 200 focus-forming units (FFU) of MPXV were prepared by diluting the viral stock with D2 medium. Sera dilutions were mixed with the MPXV in the presence or absence of 10% guinea pig serum as a source of complement (Bersee, BM361Y) and incubated at 37°C for one hour. The mixture was then added to the plated Vero E6 cells. After 24 h incubation, cells were treated with paraformaldehyde (4%) for fixation, followed by permeabilization with 0.2% Triton X-100 and blocked with 1% BSA buffer. For the staining of the infected cells, cells were incubated with anti-VACV-polyclonal antibodies (Abcam, ab35219) with a dilution of 1:8000 dilution, followed by incubating with HRP-labeled goat anti-rabbit secondary antibody (Jackson ImmunoResearch, catalog 111-035-114). Finally, KPL TrueBlue substrate (Seracare Life Science, 5510–0030) was added to each well and incubated for 10 min at RT. The plate was then washed and imaged. Foci were quantified using ELISPOT reader (Cellular Technology Ltd). GraphPad Prism 9.0 was used to analyze the FFA data. VACV microneutralization test was performed similar to MPXV neutralization assay with minor modification and Vero E6 cells were plated and used for VACV neutralization assay.

Detection of MPXV-specific neutralization antibodies by PRNT

To assess the neutralizing activity of plasma against MPXV, Vero 81 cells were seeded at a density of 2×10^5 cells per well in 12-well plates and incubated overnight. Heat-inactivated plasma samples were serially diluted 2-fold in D2 medium and mixed 1:1 with a standardized MPXV suspension. The plasma–virus mixtures were incubated at 37°C for 1 h to allow antibody–virus interaction, then added to the monolayers and incubated for an additional 1 h at 37°C. Following adsorption, cells were overlaid with 0.8% carboxymethylcellulose in DMEM supplemented with 2% fetal bovine serum. After 72 h of incubation, overlays were removed, and plaques were visualized by staining with 0.1% crystal violet. Neutralization activity was calculated as the percentage reduction in plaque number relative to virus-only controls using GraphPad Prism 8 (GraphPad Software Inc.).

Antibody depletion experiments

To selectively deplete sera of antibodies recognizing specific surface proteins of MPXV or VACV, plasma samples were incubated with recombinant proteins corresponding to either IMV- or EEV-associated antigens. The panel of proteins included MPXV H3, E8, B6, M1, A35, and A29, as well as the homologous VACV proteins H3, B5, L1, A33, and A27. In detail, a volume of serum containing one EEV-specific $PRNT_{50}$ or IMV-specific $FRNT_{50}$, respectively, was incubated with individual recombinant proteins at final concentrations ranging from 1 to 4.8 μg/mL at 37°C for 1 h with gentle agitation, as previously described.[43,44] Following depletion, residual neutralizing activity was quantified in duplicate and compared with matched untreated serum from the same donor. The percentage reduction in neutralizing activity was calculated relative to untreated controls. Based on optimization experiments, protein concentrations of 1 μg/mL (IMV) and 4.8 μg/mL (EEV) were selected for subsequent assays (Figure S7). Antibody depletion assays targeting EEV membrane proteins were evaluated using PRNT, whereas those targeting IMV membrane proteins were assessed using FRNT.

Immune plasma treatment and MPXV infection of mice

BALB/c mice, typically females aged 6–8 weeks and weighing approximately 18–20 g, have been widely employed in MPXV infection models and antiviral efficacy studies.[39,45–48] Here the immune sera collected from VACV vaccinated individuals, MPXV infection cases and convalescents were heat-inactivated for 30 min at 56°C. BALB/c mice received 200 μL per mouse via intravenous route. Four hours later, the recipient mice were challenged with 1×10^6 FFU of MPXV (clade IIb). The control group received PSB and plasma from the unvaccinated donors (<42 group) followed by the same MPXV challenge dose. Four days after challenge with MPXV, mice from each group were sacrificed, and the lung was harvested. The tissues were homogenized. Following centrifugation, the supernatants were collected and viral titer was determined by digital PCR. In parallel, lungs were fixed in zinc formalin, embedded in paraffin, tissue sections were stained with hematoxylin and eosin for examination.

MPXV DNA detection by digital PCR

Lung viral titers were quantified from homogenized lung tissues using digital PCR. Viral DNA was extracted from clarified supernatants of lung homogenates with a commercial viral DNA extraction kit (Qiagen, Hilden, Germany), according to the manufacturer's instructions. Digital PCR was then performed using the mpox Virus Nucleic Acid Detection Kit (TargetingONE Corporation, cat. no. 13500). Reactions were prepared following the manufacturer's protocol and run on a TargetingONE digital PCR system. Viral copy numbers were automatically calculated using the instrument's analysis software and expressed as copies per mL of lung homogenate. Negative controls and positive controls provided in the kit were included in each run to validate assay performance.

QUANTIFICATION AND STATISTICAL ANALYSIS

All statistical analyses were performed using GraphPad Prism 8. Correlations among ELISA binding titers, MPXV $FRNT_{50}$, VACV $FRNT_{50}$, and donor age were assessed using two-tailed, nonparametric Spearman correlation tests, with correlation coefficients (r) and p values shown in the figures. Statistical significance was assessed using one-way ANOVA with multiple comparisons tests. Error bars represent mean ± SD, p-values are displayed as ns for $p > 0.05$, *$p < 0.05$, **$p < 0.01$ and ***$p < 0.001$. PCA and visualization were performed using R version 4.4.1 and RStudio. Data were normalized prior to analysis, utilizing the 'FactoMineR', 'factoextra', and 'ggplot2' packages (Figure 4). Statistical methods specific to individual experiments are detailed in their corresponding sections.

Cell Reports Medicine, Volume *6*

Supplemental information

Comparative humoral profiles in mpox cases, survivors, and vaccinated individuals reveal correlates of protection against MPXV

Yanqun Wang, Lu Zhang, Lijuan Zhou, Jiantao Chen, Zhaoyong Zhang, Tiantian Wu, Peilan Wei, Airu Zhu, Ruoxi Cai, Jingjun Zhang, Zhiwei Lin, Canjie Chen, Yuanyuan Zhang, Qier Zhong, Jing Sun, Yongxia Shi, Jingxian Zhao, Jun Dai, Pengzhe Qin, and Jincun Zhao

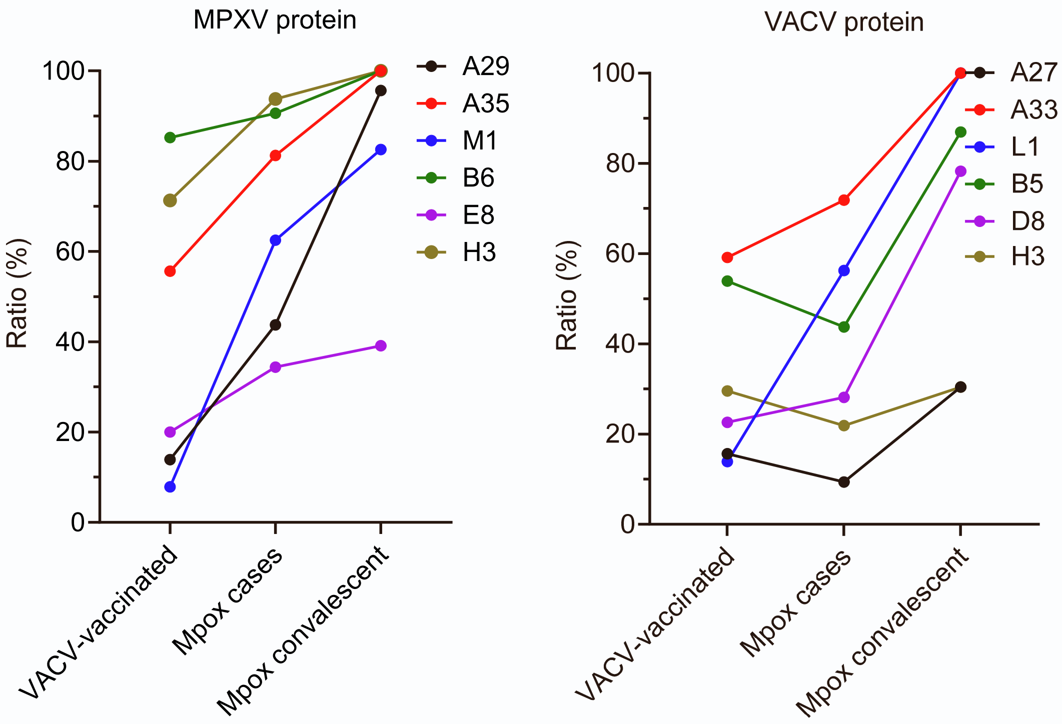

Figure S1. Comparisons of seropositivity rates of binding IgG against different orthopoxvirus antigens between mpox cases, convalescents and VACV vaccinated individuals. Related to Figure 1.

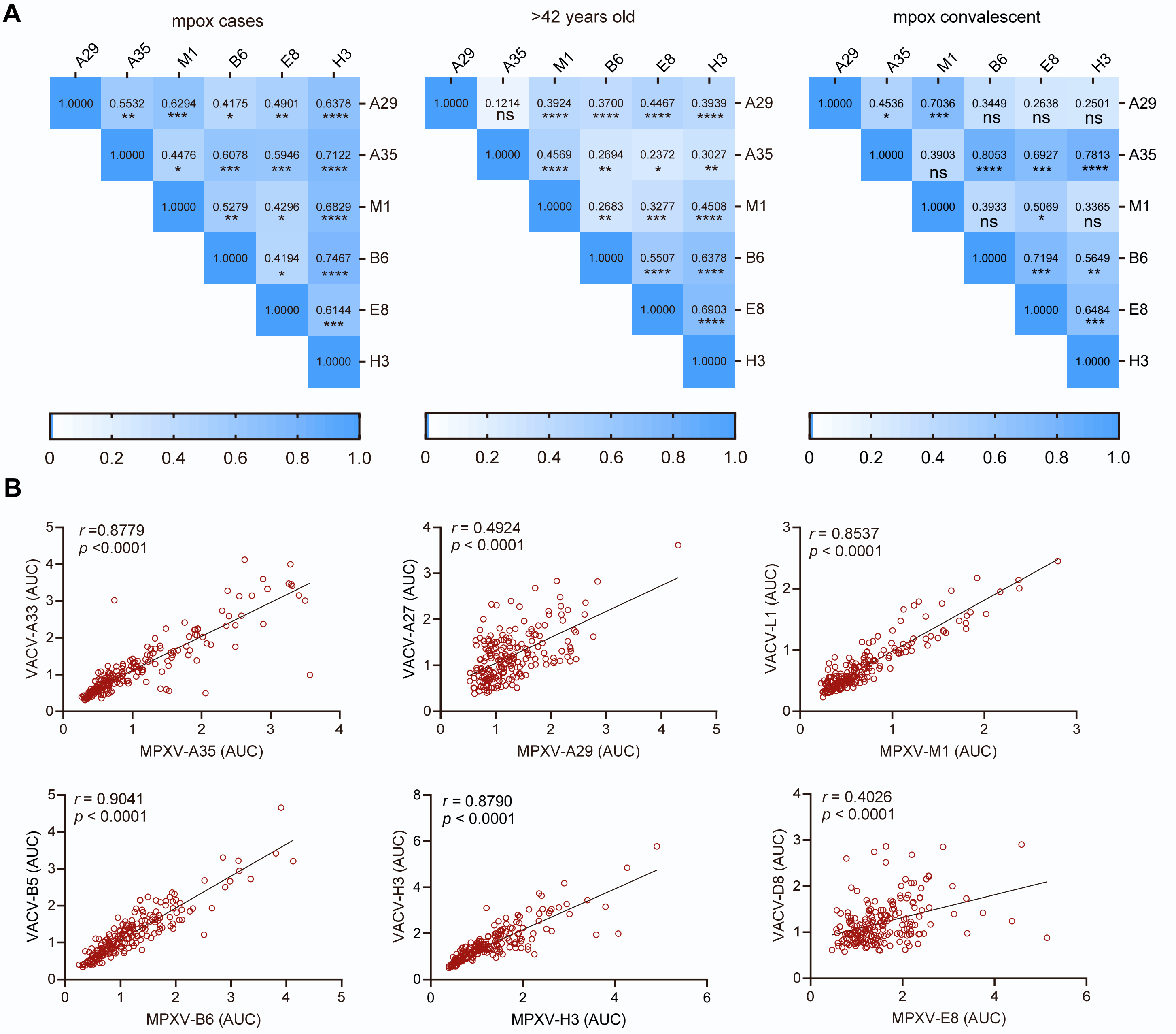

Figure S2. Correlation analysis of MPXV- and VACV-specific protein binding IgG response. Related to Figure 1. **(A)** Correlation matrix of IgG antibody response against different MPXV antigens (A29, A35, M1, B6, E8 and H3) in mpox cases, convalescents and VACV-vaccinated individuals (>42 years old). Color scale represents the value of the correlation between two variables. **(B)** Correlation analysis of MPXV (A29, A35, M1, B6, E8 and H3) and VACV (A33, A27, L1, B5, D8 and H3) antigens binding IgG response among all participants. Spearman's correlation coefficients and *p* values are shown.

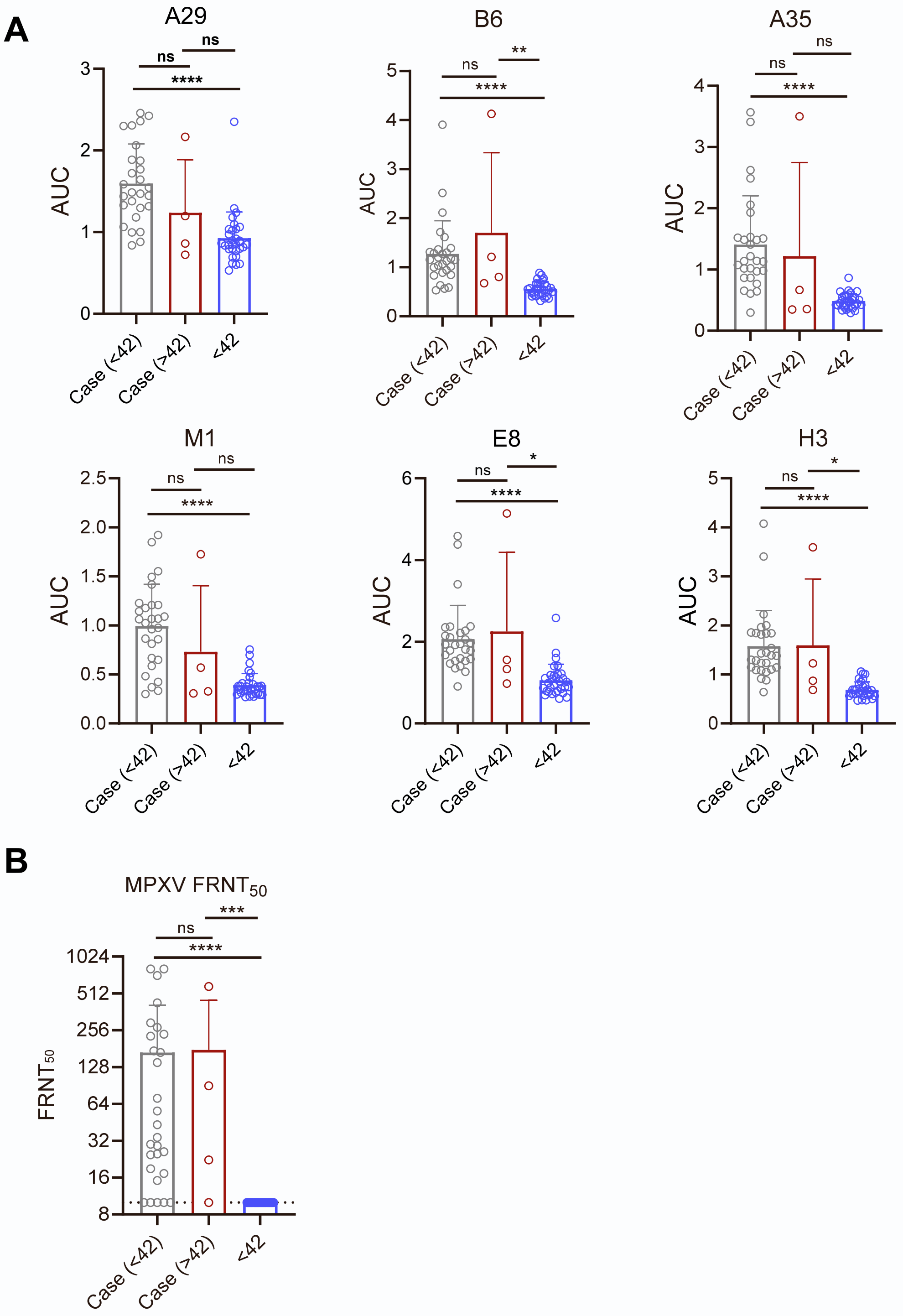

Figure S3. Pre-existing humoral immunity to orthopoxvirus does not impact the MPXV antibody response based on the limited participants. Related to Figure 1. (A) Comparisons of seropositivity rates of binding IgG against different MPXV antigens (A29, A35, M1, B6, E8 and H3) between mpox cases with and without pre-existing VACV antibody. **(B)** Comparisons of neutralizing antibody titer against authentic MPXV between mpox cases with (case with age >42) and without pre-existing VACV antibody (case with age <42). Statistical significance was assessed by one-way ANOVA with multiple comparisons in (A) and (B). Error bars represent mean ± SD, *p*-values are displayed as ns for $p > 0.05$, $*p < 0.05$, $**p < 0.01$ and $***p < 0.001$.

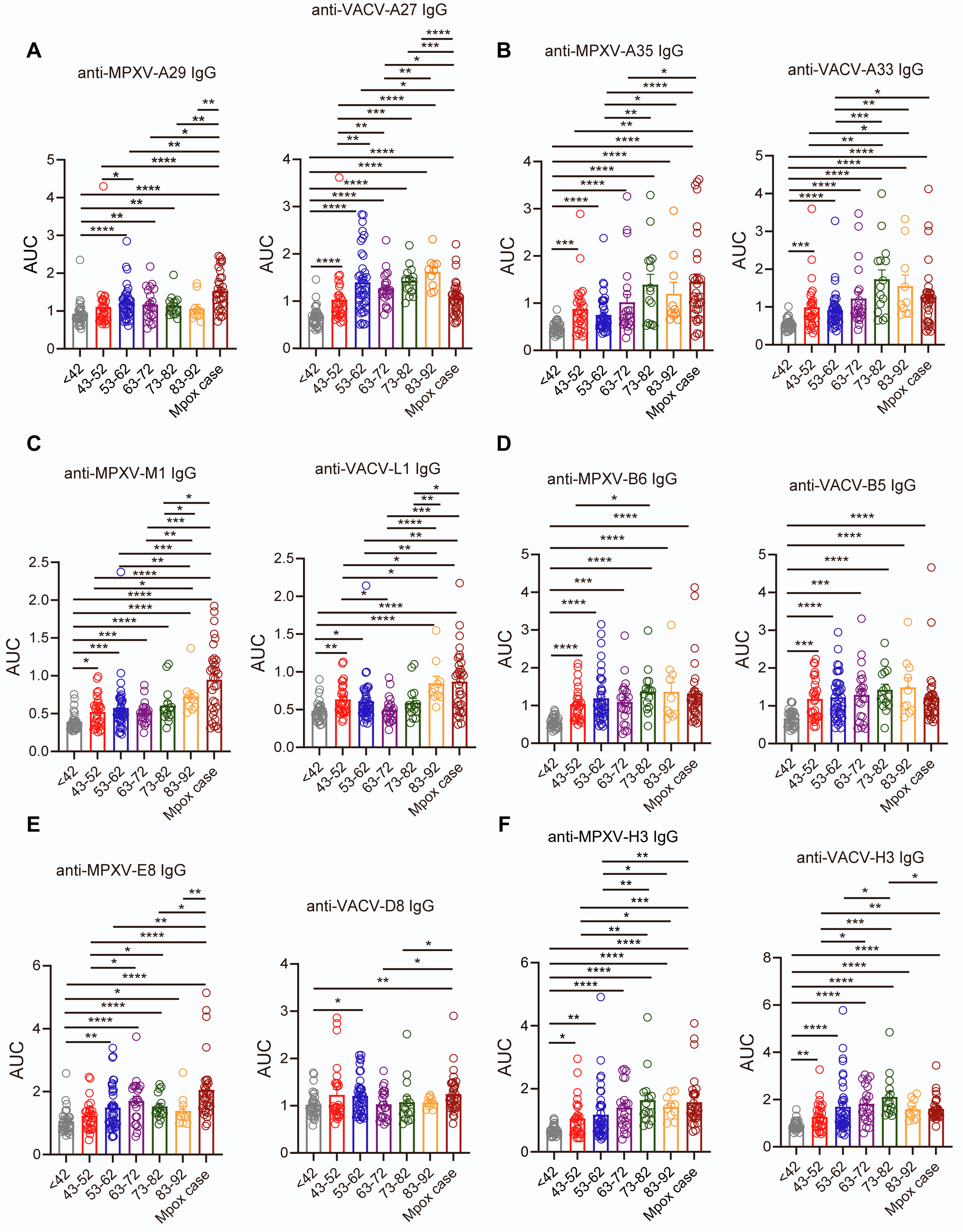

Figure S4. The distribution of antibody titers against multiple orthopoxvirus antigens (A-F) was analyzed in VACV-vaccinated donors, stratified into five age groups by decade of birth. Related to Figure 2. VACV-unvaccinated individuals and MPXV-infected cases were included as controls

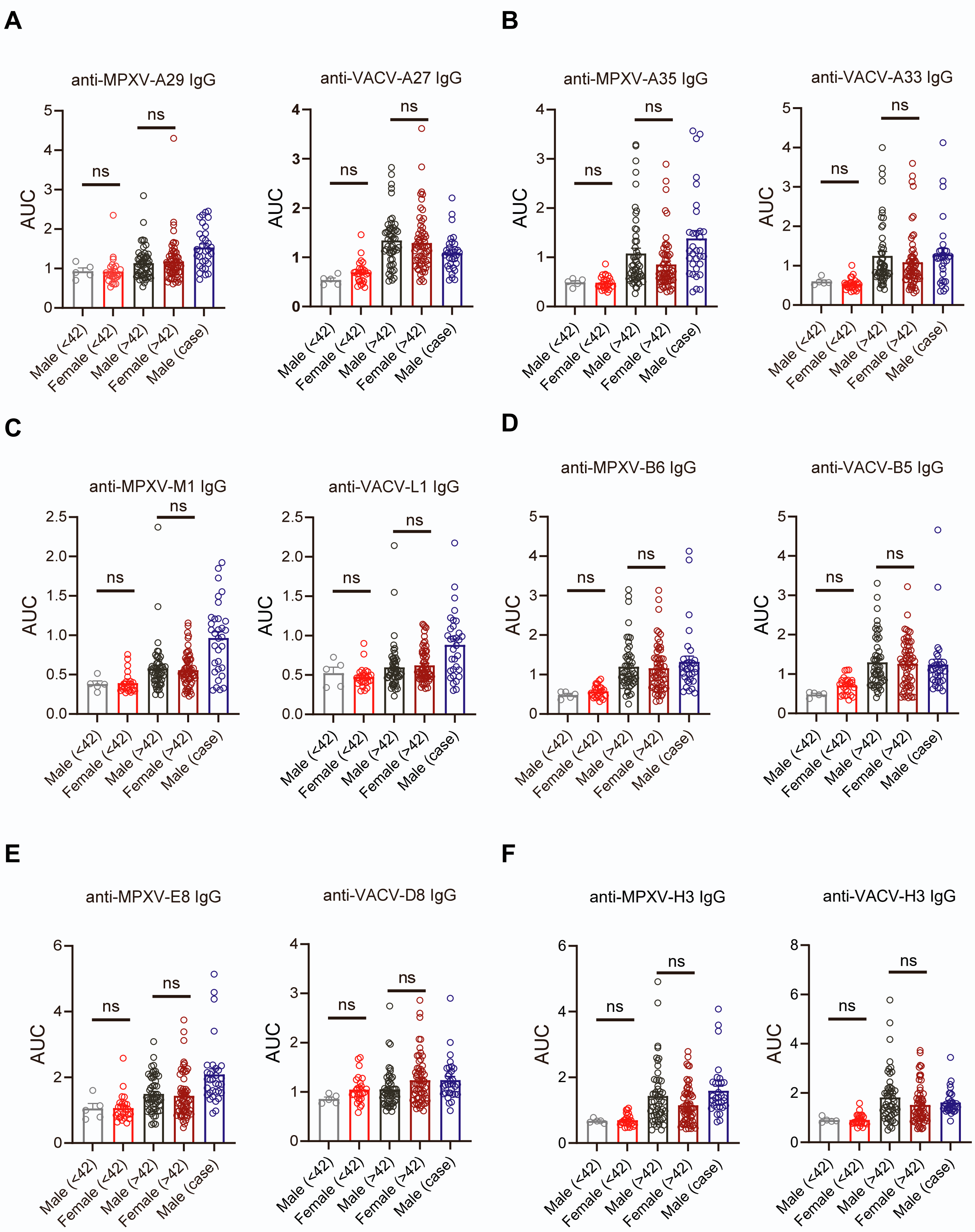

Figure S5. Relationship between binding antibodies against orthopoxvirus antigens and gender. Related to Figure 2. (A-F) Comparison of IgG antibody response against MPXV (A29, A35, M1, B6, E8, H3)) and VACV (A27, A33, L1, B5, D8, H3) antigens across different gender cohorts. Statistical significance was assessed using one-way ANOVA with multiple comparisons tests. Error bars represent mean ± SD, p-values are displayed as ns for $p > 0.05$, $*p < 0.05$, $**p < 0.01$ and $***p < 0.001$.

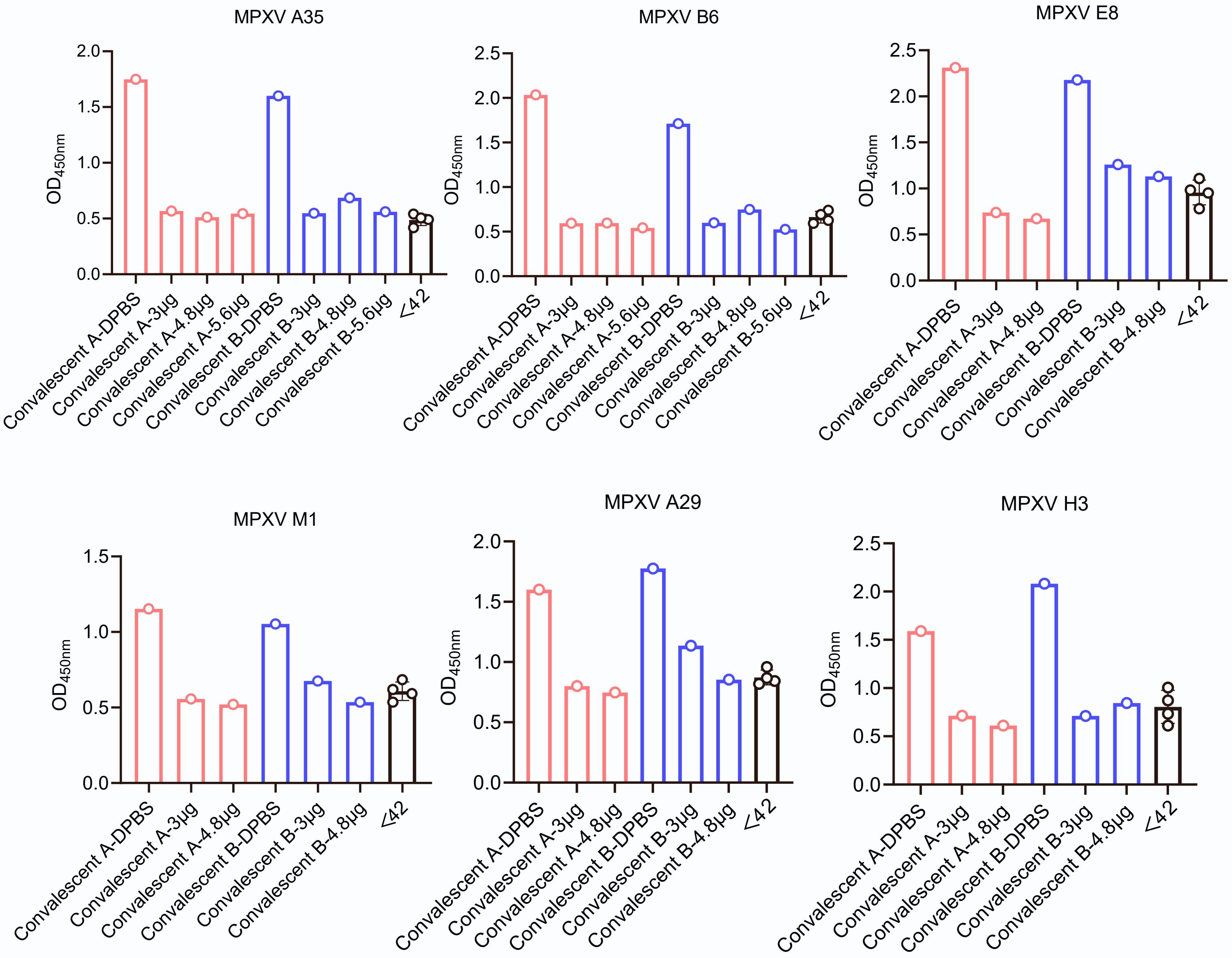

Figure S6. Quantification of plasma IgG binding to MPXV membrane proteins following competitive blocking with increasing amounts of membrane protein. Related to Figure 5. Plasma from two mpox convalescents (A and B) and four VACV-unvaccinated individuals (controls) was analyzed. Six membrane proteins (A35, B6, E8, M1, A29, and H3) were included in the assay. Data are representative of two independent experiments.

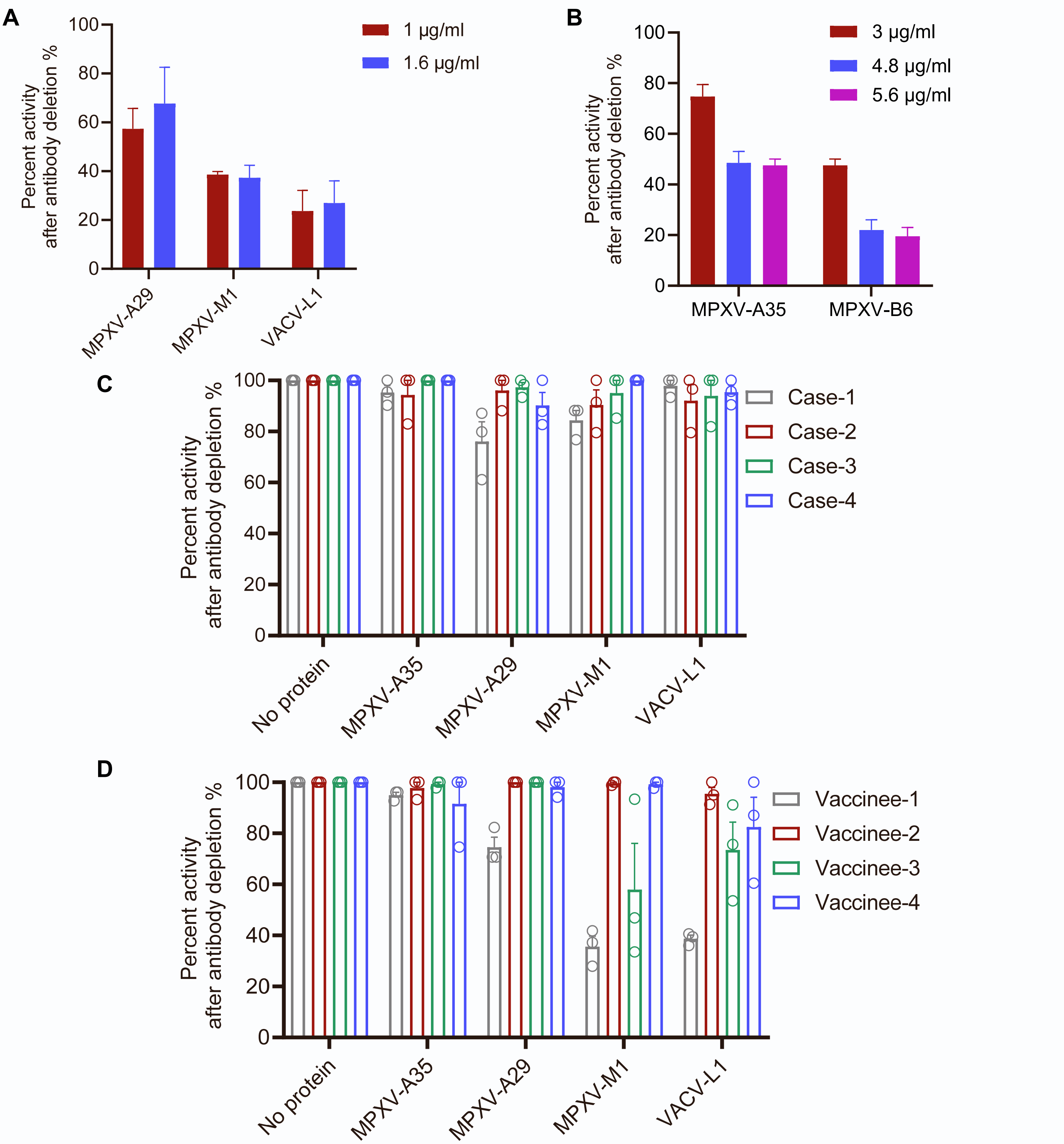

Figure S7. Optimization and application of antibody depletion assays. Related to Figure 5. (**A–B**) Optimization of antigen concentrations for antibody depletion. Residual neutralizing activity against IMV (**A**) and EEV (**B**) in human plasma following depletion with varying antigen concentrations. Based on these results, protein concentrations of 1 μg/mL (IMV) and 4.8 μg/mL (EEV) were selected for subsequent assays. (**C–D**) Application of antibody depletion assays. Residual neutralizing activity against MPXV IMV after depletion with purified A35, A29, M1, or L1 proteins in plasma from MPXV-infected individuals (**C**) and historically VACV-vaccinated individuals (**D**). Antibody depletion assays targeting IMV membrane proteins were evaluated using FRNT, whereas those targeting EEV membrane proteins were assessed using PRNT.

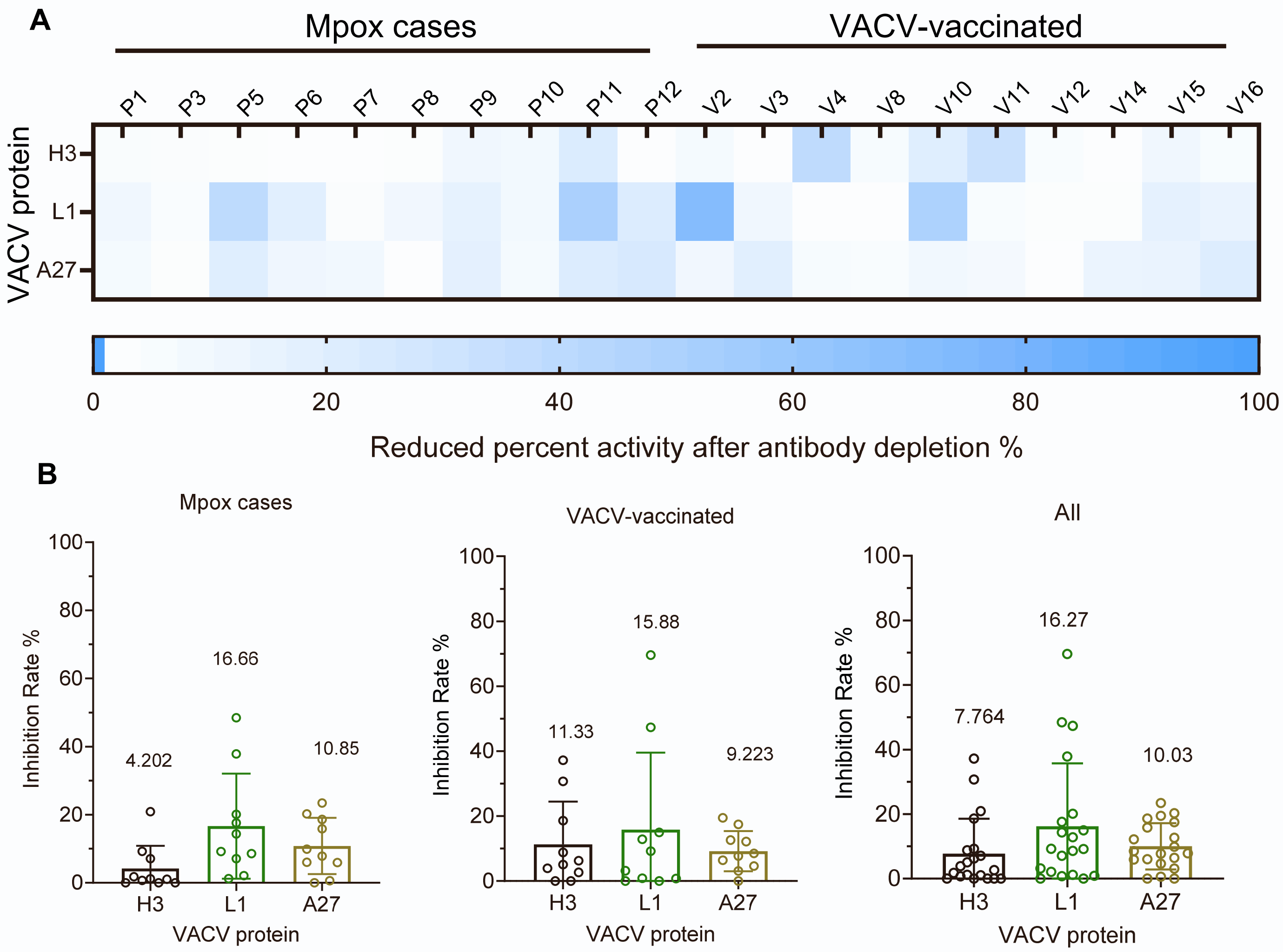

Figure S8. Quantification of neutralizing antibody responses against representative antigens of VACV by antibody depletion experiments. Related to Figure 5. (A) Heatmap of reduced neutralizing activity percentage of plasma after antibody depletion from VACV-vaccinated (>42 years old) or MPXV-infected individuals using multiple antigens of VACV (H3, L1 and A27). Donors' number was labeled in every column. Color scale represents the percentage of reduced neutralizing activity against VACV IMV. **(B)** Comparison of reduced neutralizing activity against IMV after incubation with variable VACV antigens. The average inhibition ratios are shown in the upper panel.

Table S2. Neutralizing titers against MPXV were determined from pooled plasma samples. Related to Figure 6.

MPXV $FRNT_{50}$	<42	>42	Case	Convalescent
Complement (-)	10	65.16	288.4	138.03
Complement (+)	10	173.78	401.79	325.83
